# Synth4bench: generating synthetic data for benchmarking tumor-only somatic variant calling algorithms

**DOI:** 10.3389/fbinf.2026.1858375

**Published:** 2026-07-16

**Authors:** Styliani-Christina Fragkouli, Nikos Pechlivanis, Anastasia Anastasiadou, Georgios Karakatsoulis, Aspasia Orfanou, Panagoula Kollia, Andreas Agathangelidis, Fotis Psomopoulos

**Affiliations:** 1 Department of Biology, National and Kapodistrian University of Athens, Athens, Greece; 2 Institute of Applied Biosciences, Centre for Research and Technology Hellas, Thessaloniki, Greece

**Keywords:** benchmarking, genomics, software, synthetic data, variant calling

## Abstract

**Background:**

Somatic variant calling is a key activity towards identifying genomic alterations; yet, the evaluation of the respective tools remains challenging due to the scarcity of high quality ground truth datasets. To overcome this limitation, we developed synth4bench, a synthetic data generation pipeline, which utilizes the NEAT simulator, for robust benchmarking. Using a systematic process to create distinct synthetic datasets, we thoroughly evaluated five variant callers (Mutect2, FreeBayes, VarDict, VarScan2 and LoFreq). We compared tool outputs against our synthetic ground truth across key sequencing aspects (such as depth and read length) to assess their capacities and shed light on their underlying algorithmic principles.

**Results:**

Synth4bench is an approach for evaluating tumor-only somatic variant callers that relies on a systematic definition of fully controlled ground-truth datasets. Our analysis revealed significant inconsistencies among the tool outputs and a strong dependence of caller performance on sequencing parameters. Indels remain the hardest-to-call variant type, driven by errors at low allele frequencies. Algorithmic choice is also critical; the most robust callers displayed the highest Precision in allele frequency estimation, while the most sensitive caller was best for maximizing true positive recovery. Conversely, the least suitable caller exhibited systematic errors along with the poorest overall performance.

**Conclusion:**

These findings indicate that there is not a one-size-fits-all approach; sequencing optimization together with caller selection are necessary to maximize sensitivity and reliability. Furthermore, the pronounced inconsistencies suggest that current algorithms are not yet able to capture all mutational mechanisms adequately, with the modeling of the underlying processes remaining an open challenge.

## Highlights

• **Indels:** the choice of tool should reflect the study aim, Precision prioritizing workflows can prefer LoFreq and VarScan2, whereas recall-critical prioritizing workflows can prefer VarDict which had the highest Recall values and the smallest Precision and Recall trade-off.

• **Recall and Precision:** These findings indicate that sequencing-specific optimization of the selected caller is necessary to maximize sensitivity and reliability, particularly in applications where Recall or Precision are critical. Caller should be fit-for-purpose, as there is not a one-size-fits-all approach.

• **Frequency resolution**: The results highlight the trade-off between Recall and Precision, particularly at low AF ranges where variant detection is most challenging. Callers like VarScan2 and VarDict are more suitable for variant detection at the lower end of the spectrum.

• **True variants:** callers should be selected according to study priorities: VarScan2 for maximizing TP recovery of true variants, with awareness of its mild AF underestimation, LoFreq when precise AF estimation is paramount.

• **Disagreement:** Such pronounced inconsistencies suggest that current algorithms do not yet capture mutational mechanisms adequately, indicating that fully modeling of the underlying processes remains an open challenge.

## 1 Introduction

Genomic DNA alterations are strongly implicated in the pathogenesis of diseases such as hereditary disorders and cancer. Next-generation sequencing (NGS) technologies have greatly assisted in capturing the actual genomic complexity of cancer, through the identification of thousands of somatic mutations related to a variety of human cancers (Zhang et al., 2016; Martínez-Jiménez et al., 2020). However, the accurate detection of somatic variants, particularly those present at low frequencies, remains a challenge due to limitations in sequencing technologies, analytical pipelines and the application of stringent filtering criteria. Consequently, low-frequency variants may be overlooked, potentially limiting the comprehensive characterization of genomic landscapes in disease studies and clinical settings. (Shin et al., 2017; Olson et al., 2023).

To date, a large series of bioinformatics tools are available for the detection of all different types of mutations, including single nucleotide variants (SNVs), as well as small insertions and deletions (indels). These tools can be classified according to both their application domain and methodological framework. Germline callers are primarily designed to detect inherited variants occurring at relatively high allele frequencies, whereas somatic callers target acquired mutations that are often present at low frequencies and are therefore more difficult to distinguish from sequencing artifacts. Somatic variant callers can be further divided into matched tumor–normal approaches, which utilize a paired normal sample for artifact and germline filtering and tumor-only approaches, which rely solely on tumor sequencing data. Furthermore, variant callers use different computational strategies, including haplotype-based local assembly methods, probabilistic frameworks and quality-score/statistical models. For example, Mutect2 and FreeBayes incorporate haplotype-based local assembly approaches, LoFreq relies on statistical modeling, while VarScan2 and VarDict primarily use heuristic and filtering strategies. These methodological differences can substantially affect variant detection performance, particularly in challenging low-frequency settings. The variant callers evaluated in this study represent different methodological categories commonly used in tumor-only somatic variant detection.

Although several benchmarking initiatives and reference resources, including Genome in a Bottle (GIAB), the ICGC-TCGA DREAM Challenge and the SEQC2 Consortium, have contributed substantially toward the standardization of variant calling evaluation, benchmarking practices remain heterogeneous across studies due to differences in truth sets, experimental designs, sequencing conditions and performance metrics (Olson et al., 2023; Li et al., 2024; Guille et al., 2024; Xiang et al., 2023; Ha et al., 2023; Kumaran et al., 2019; Sandmann et al., 2017; Krøigård et al., 2016; ICGC-TCGA DREAM Somatic Mutation Calling Challenge participants et al., 2015; Kim and Speed, 2013; Cai et al., 2016; Xu, 2018; Sandmann et al., 2018). Furthermore, in the case of variants identified at a low allele frequency (AF) (i.e., ≤10%), robust identification becomes even more demanding, requiring higher levels of sensitivity across analytical workflows (Xiang et al., 2023; Federici and Soddu, 2020).

This unresolved issue is reflected in the limited agreement among different variant callers, particularly for low-frequency variants that are inherently more difficult to detect (Ha et al., 2023). Even small discrepancies between variant callers can lead to relevant variants being missed or inconsistently reported, thereby affecting downstream analyses, result reproducibility and potentially clinical interpretation. To this date, several publications have dealt with the benchmarking and comparison of relevant algorithms (Sandmann et al., 2017; Krøigård et al., 2016; He et al., 2021). To improve reliability, variant calling pipelines have been refined with new methods and strategies designed to reduce discordant and inconsistent results.

Furthermore, the assessment of variant calling capacity has long been hindered by the scarcity of high-quality datasets that could serve as ground truth (ICGC-TCGA DREAM Somatic Mutation Calling Challenge participants et al., 2015; Kim and Speed, 2013). The implementation of such datasets would render the process of benchmarking more efficient and robust (Xu, 2018; Majidian et al., 2023). A relevant solution concerns the use of synthetic data from the *in silico* simulation of synthetic genomics, which has evolved over the last 30 years (Engle and Burks, 1993) alongside sequencing technologies (Ha et al., 2023; Alosaimi et al., 2020).

In this work, we present synth4bench, a bioinformatics pipeline designed to systematically observe and compare the behavior of variant calling algorithms. Our primary objective was to evaluate the detection ability of callers under controlled experimental conditions. To this end, synthetic NGS datasets were generated using the NEAT simulator, while synth4bench was responsible for the orchestration of dataset generation, controlled manipulation of sequencing parameters, construction of reproducible benchmarking datasets, definition of ground-truth variants and downstream comparative evaluation of variant calling performance. By systematically varying key sequencing characteristics, including coverage and read length, the framework enables the investigation of caller-specific sensitivities and performance trends, particularly in low-frequency somatic variant detection settings. The coverage range (300×–5000×) was selected to reflect high-depth, panel-like sequencing scenarios where low AF detection is critical, particularly in tumor-only settings. This depth spectrum allows systematic evaluation of caller performance as signal-to-noise ratios increase. Lower-coverage regimes typical of WES (whole-exome sequencing) or WGS (whole-genome sequencing) were not included, as the study focused specifically on ultra-deep configurations relevant to low AF detection. A subset of variants was considered as “true”, enabling performance assessment based on the ability of each algorithm to detect and accurately quantify these variants. Rather than attempting to reproduce the full biological complexity of sequencing data, synth4bench was developed as a technical stress-testing pipeline that allows direct, structured, and reproducible comparison of algorithmic behavior. Our results demonstrate that this approach provides valuable insight into caller decision patterns and performance trends across different NGS parameter configurations.

## 2 Materials and methods

**FIGURE 1:**
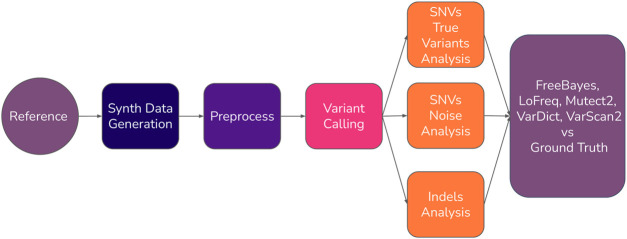
Schematic representation of synth4bench. The circles represent the input and output files, while the rectangles represent the in-between processes. Three different analyses (two for SNVs true variants and noise, and one for indels) follow after the step of variant calling.

Our pipeline takes as input a reference human genome, the NGS-related parameters (as set by the user) and outputs the comparison of results between each variant caller and the ground truth. The in-between steps include the preprocessing required before moving to the variant calling process, as well as the downstream analysis of reported variants as depicted in Figure 1. The comparison concerns results from different analyses, which were implemented in a way to adequately investigate both SNVs and indels. The complete methodology is detailed in Table 1. All steps and respective tools, as implemented in synth4bench for data generation, pre-process, variant calling and benchmarking. Our pipeline can be dynamically adapted; new callers can be added in the future.

**TABLE 1:** Complete set of computational steps of synth4bench.

Synthetic data generation steps	Tool
Generation of individual synthetic ground truth files	NEAT
merge individual bam ground truth files	samtools merge
Index individual vcf ground truth files	Bcftools index
merge individual vcf ground truth files	Bcftools merge
Normalize merged vcf ground truth file	Bcftools norm

Common pre-process steps	Tool
Sort merged bam ground truth file	samtools sort
Clean merged bam ground truth file	samtools view
Alignment ground truth statistics	samtools flagstat
Add read group tags in a merged bam ground truth file	samtools addreplacerg
Compute ground truth coverage per position	samtools depth
Index all bam ground truth files	samtools index
Get low-level information about data at each position for all bam ground truth files	Bam-readcount

Mutect2 specific steps	Tool
Call Mutect2 to perform variant calling	gatk Mutect2
Normalize Mutect2 vcf file	Bcftools norm

FreeBayes specific steps	Tool
Call FreeBayes to perform variant calling	FreeBayes
Modify header of FreeBayes vcf file	Bcftools reheader
Normalize FreeBayes vcf file	Bcftools norm

LoFreq specific steps	Tool
Add indel qualities to ground truth merged bam file	LoFreq indelqual
Call LoFreq to perform variant calling	LoFreq call
Modify header of LoFreq vcf file	Bcftools reheader
Normalize LoFreq vcf file	Bcftools norm

VarScan2 specific steps	Tool
Get “pileup” textual format from ground truth merged bam file	samtools mpileup
Call VarScan2 to perform variant calling	varscan pileup2cns
Convert VarScan2 tsv file to vcf file	Extra python script
Normalize VarScan2 vcf file	Bcftools norm

VarDict specific steps	Tool
Call VarDict to perform variant calling	vardict
Convert VarDict txt file to vcf file	var2vcf
Modify header of VarDict vcf file	Bcftools reheader
Normalize VarDict vcf file	Bcftools norm

Benchmarking steps	Tool
Call ground truth and callers’ files to compare	Synth4bench R scripts

### 2.1 Generate the synthetic ground truth datasets

Due to its central role within our pipeline, the generation of synthetic human genomics data was a task of high importance. The selected simulator was NEAT (NExt-generation sequencing Analysis Toolkit) (Stephens et al., 2016; Alosaimi et al., 2020) due to its flexibility and broad range of configurable parameters, which offers extra user control over the experiment, compared to other simulation frameworks (Milhaven and Pfeifer, 2023). Also, NEAT enables the modeling of sequencing and mutation characteristics relevant to benchmarking low-frequency somatic variant detection, including customizable mutation models, sequencing error profiles and GC-content bias. In this study, the default models provided by the software were utilized, as only the reference genome was used as input and no sequencing dataset was available for the construction of custom error models. In addition, NEAT directly produces aligned sequencing data in BAM format, thus facilitating controlled condition benchmarking analyses.

Although synth4bench uses NEAT for data simulation, its purpose goes far beyond read generation. It provides a fully integrated benchmarking pipeline that systematically varies sequencing parameters, constructs controlled low AF scenarios and performs structured multi-metric evaluation of SNVs and indels against a defined ground truth. Unlike using NEAT alone, synth4bench delivers a reproducible, end-to-end environment for standardized and comparative assessment of tumor-only somatic variant callers. Its value lies in enabling controlled and systematic benchmarking rather than simple simulation.

The simulated mutation set is generated during the data generation step, where mutations are introduced according to user-defined simulation parameters following NEAT’s documentation. A list of parameters and a short description are presented in Supplementary Material Section S1. The reference human genome used during the data generation was hg38 (GRCh38), and a defined seed parameter ensured reproducibility. All seed values used for each dataset were stored in a database together with the corresponding simulation settings and are available in Supplementary Material Section S2, enabling transparent and fully traceable reconstruction of the ground truth for every benchmark experiment.

One of the main aims of this work was to better understand the behavior of variant callers. To this end, it was crucial to test their response to data with varying values and thus we constructed a feature space by using ranging values for given characteristics. Coverage, which refers to the count of reads covering each base of the sequenced DNA, is a feature that plays an important role when dealing with NGS-related errors. One approach to ensure accurate base calling at each given position was by increasing the coverage (Sims et al., 2014).

In order to assess in-depth the behavior of variant callers, the coverage values that were chosen ranged from 300x to 5000x, as shown in Supplementary Material Section S1. Expanding to additional coverage regimes would significantly increase computational complexity. The design should therefore be interpreted as a focused, panel-oriented benchmark rather than a universal sequencing evaluation.

Another chosen feature was the read length of NGS data; lengths ranging from 50 base pairs (bp) to 300bp were put to test. This approach was applied to cover all possible scenarios for a given fragment (in our case with a mean length of 300bp and standard deviation of 30bp).

Grid experiments were performed to cover all the constructed feature space as shown in Table 2. Since we worked with a human reference genome, the ploidy parameter was set to 2 and the average mutation rate was fixed to 0.1 to generate sufficiently dense variant sets for robust benchmarking across callers and sequencing conditions. The rationale for a fixed mutation rate, along with a simplification of diploid approach, is to not replicate the mutational burden of a specific tumor type, but to ensure adequate SNV and indel representation under controlled conditions, making synth4bench a diploid technical benchmark rather than a realistic tumor genome modeling pipeline. Ultimately, one of the key aims of synth4bench is to position itself as a technical solution for reproducible benchmarking activities in the field of variant calling. Variant AFs emerged from the merging strategy, where 100% AF variants were proportionally diluted to create a structured and reproducible AF spectrum. Thus, variant counts and AF distributions reflect a technical benchmarking design rather than biological tumor heterogeneity. The pipeline prioritizes methodological control and reproducibility to isolate caller-specific performance characteristics.

**TABLE 2:** Values per file type along with an example.

	Coverage	Allele frequency
**Individual BAM file**	x (e.g., x=100 )	f (e.g., f=40% )
**Merged BAM**	X=10·x (e.g., X=1000 )	F=f/10 (e.g., F=4% )

The simulated mutation set was generated internally using NEAT with predefined parameters to ensure controlled and reproducible ground truth construction. A total of 29 datasets were created with all parameters and seeds recorded for transparency. Variant AF emerged from the merging strategy, where variants at 100% AF in individual files were proportionally diluted (1, 2, 4, or 10 merged files), producing a structured AF spectrum.

Synthetic datasets were generated from the TP53 locus within the hg38 reference genome for computational efficiency. Mutations followed the simulator’s model within the hg38 framework, with genomic position not influencing the technical benchmarking objective.

Finally, the *bam-readcount* package was implemented to obtain low-level information for all positions in the ground truth Binary Alignment Map (BAM) files, thereby enhancing the benchmarking process.

### 2.2 Design the benchmarking

Accurately mapping reads to the reference genome by using alignment tools is important when identifying genetic variants. However, it is known that the choice of a variant caller exerts a greater influence on the detection of variants (Xu, 2018). Thanks to the fact that NEAT produces aligned reads, we were able to skip the alignment step and focus merely on the variants’ detection, generated directly in the BAM format.

**FIGURE 2:**
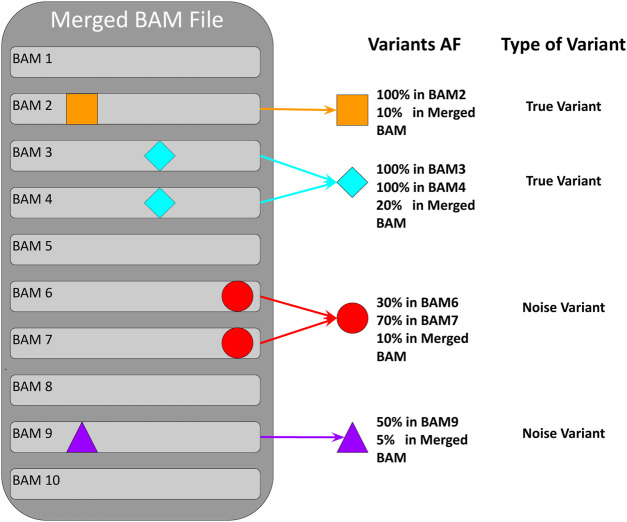
Schematic overview of the method used to define True Variants. Each horizontal bar represents an independently simulated BAM file. Colored symbols indicate variants introduced during simulation. Variants that occur at f=100% AF within an individual BAM file are defined as True Variants (e.g., orange square in BAM2 and cyan diamonds in BAM3 and BAM4). In contrast, variants that occur at lower than f=100% frequencies in individual BAM files (e.g., Red circle at f=30% AF in BAM6 and f=70% AF in BAM7 or purple triangle at f=50% AF in BAM9) are treated as noise (i.e., non True Variants). During the merging step, all 10 BAM files are combined, causing variants that overlap at the same genomic position, to become proportionally diluted in the final merged BAM file (e.g., cyan diamond becomes F=20% AF in the merged BAM and the red circle becomes F=10% AF) according to Equation 2.

To emphasize low AF variants, all BAM and Variant Call Format (VCF) files from independent runs were merged into a single ground truth file, as shown in Figure 2. Low AF variants were generated by merging independent simulated BAM files, where variants present at 100% AF in single files became proportionally diluted after merging (e.g., 1/10 → ∼10% AF). This approach enabled precise AF control while preserving clear ground truth, allowing systematic evaluation of caller performance in low-frequency scenarios and is discussed in detail in Section 2.2.2. During the preprocessing phase, some steps were common for all callers, such as indexing. On the other hand, each variant caller has its own demands. All necessary common steps were incorporated in our pipeline using SAMtools and bcftools (Danecek et al., 2021) and can be found in Table 1.

Our primary goal in this study was to evaluate the technical performance of variant callers. To maintain a consistent and unbiased benchmark, variant annotation was not included. This approach focuses on methodological aspects, without considering biological context such as genomic regions or mutation hotspots, which remain important but are beyond the scope of this analysis.

#### 2.2.1 Choosing variant callers

As previously noted (Xu, 2018), the nature of variants can largely determine the choice of variant caller. In this study, we focused on popular variant callers, capable of detecting both SNVs and indels (Olson et al., 2023; Guille et al., 2024; Xiang et al., 2023; Sandmann et al., 2017; Krøigård et al., 2016; Cai et al., 2016; Xu, 2018; Sandmann et al., 2018). Furthermore, only primary and openly available algorithms were considered. Based on these criteria, the five popular variant callers selected for benchmarking were Mutect2 from GATK 4.3.0.0 (Cibulskis et al., 2013), FreeBayes 1.3.6 (Garrison and Marth, 2012), LoFreq 2.1.5 (Wilm et al., 2012), VarScan2 2.4.6 (Koboldt et al., 2012) and VarDict 1.8.3 (Lai et al., 2016), all installed via a conda environment for easier implementation. Ensemble methods were not included in our study. A candidate that was ultimately left out was Strelka2 (Kim et al., 2018), since it operates only in matched tumor-normal DNA samples. All selected algorithms accept BAM files as input and produce VCF files as output. The sole exception was VarScan2, which required a pile up file as input while additional processing was needed to convert its output from Tab-Separated Values (TSV) format to VCF. All variant callers were run according to the indicated best practices (Best practises VarDict, 2025; Best practises Freebayes, 2025; Best practises VarScan, 2026; Best practises LoFreq, 2026; Best practises Mutect2, 2025).

#### 2.2.2 SNVs independent analyses of true variants and noise

In this study, we aimed to systematically evaluate the detection ability of variant calling algorithms under controlled experimental conditions. To achieve this, a predefined subset of variants was considered as “true” and caller performance was assessed based on their capacity to detect and accurately quantify these variants. Rather than attempting to model the full biological complexity of sequencing data, our methodology was designed to function as a technical stress test, enabling direct comparison of algorithmic behavior in a structured and reproducible setting.

To achieve this, we generated a number N of individual BAM files with the same NGS characteristics. These individual BAM files were then combined into a final merged BAM file, yielding a final coverage X equal to:

X=N·x, (1)

where x denotes the coverage of the individual files. Taking into account that the AF of a variant in one of the individual BAM files was f , then the new AF, F of the same variant in the merged BAM file, becomes:

$$F=\frac{f}{N\;}.$$ (2)

The complete mathematical proof and reasoning behind Equation 2, can be found in Supplementary Material Section S12. After running a number of experiments to determine the number of individual BAM files N , we concluded that the optimal value in our case was N=10 because it assisted in reducing sufficiently the overall AFs. Hence, Equation 1 becomes X=10x and Equation 2 becomes $F=\frac{f}{10}$ . Although the theoretical dilution of a variant present at 100% AF in one of ten equally covered individual BAM files would be approximately 10% in the final merged BAM, the observed ground-truth AF occasionally deviated from this expectation. This occurs because NEAT simulates a target average coverage, resulting in variation in the depth of individual BAM files. Consequently, variants originating from lower-depth BAMs contribute proportionally fewer supporting reads to the merged dataset, leading to ground-truth AF values that may fall below the theoretical 10% expectation.

Conceptually, this is equivalent to having a predefined list of inserted variants; however, instead of supplying a static list, the list of true variants is generated dynamically within each experiment through controlled simulation and merging.

As illustrated in Figure 2, only variants present at f=100% AF in individual BAM files were considered True Variants. During the merging process, these variants could be proportionally diluted (according to Equation 2) to lower AF values in the final merged BAM file (e.g., f=100% in one out of ten BAM files resulting in F=∼10% AF). All other SNVs present in individual BAM files (i.e., those that were not present at f=100% AF) were treated as noise even in cases of considerable AF (like the example of the variant with 50% AF in the individual BAM file). It should be noted that the term “noise” is used here as a benchmarking category. Specifically, variants present at 100% AF were designated as the predefined true variant set and tracked throughout the dilution process, while all remaining variants were grouped into the noise analysis. This design allowed us to evaluate caller behavior in the presence of background variant signal that was not part of the predefined true variant set. In cases where variants originating from different individual BAM files at the same genomic position and represented the same nucleotide change, their frequencies were combined in the merged BAM file. A summary of the values, along with an example can be found in Table 2.

#### 2.2.3 Indels independent analysis

As previously discussed (Xu, 2018; Sandmann et al., 2018; Tan et al., 2015) detection of indels poses a greater challenge, thus hindering the comparison of their calling. To address this challenge, we followed the practice of indel normalization with BCFtools (Danecek et al., 2021) each variant was normalized based on two conditions; reporting was based on the smallest base position possible (i.e., left-aligned) and the length of each reported variant was the shortest possible (i.e., parsimonious).

As also discussed in (Sandmann et al., 2018) these more complex variants are inconsistently reported by variant callers. For this reason, our indel analysis included some extra steps regarding their normalisation. For example, a deletion that was reported as T > -A in some callers was converted to TA>T to maintain the same reporting method, while calibration of the POS was also performed where needed.

**FIGURE 3:**
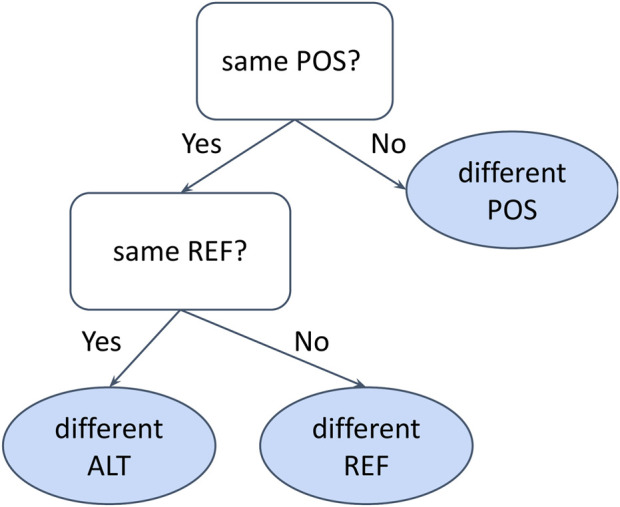
Decision tree for mismatched indels categorization. At each node, variants were compared for identical genomic position (POS), reference allele (REF), and alternate allele (ALT). Variants were classified as differing in POS, REF, or ALT depending on the outcome of these comparisons.

Furthermore, we added another layer of granularity to indels that were misidentified by examining the difference in better positioning the mismatch between the caller output and the ground truth. A decision tree was used for these steps, depicted in Figure 3, in order to categorize each indel as diff POS, diff REF and diff ALT if they were mismatched in terms of genomic position, reference allele and alternate allele column of the vcf, respectively.

Our benchmark focused on short insertion and deletion events, corresponding to the variant class targeted by the evaluated small-variant calling algorithms. The dataset included a variety of sequence patterns, ranging from repeated occurrences of a single nucleotide to events involving combinations of multiple nucleotide types, thereby providing a diverse set of insertion and deletion configurations for benchmarking purposes.

#### 2.2.4 Define classes and metrics

To systematically evaluate the performance of variant callers, we introduced a set of metrics designed to assess their accuracy and reliability in detecting both SNVs and indels. We considered three classes of variants; **False Negative** (FN), **True Positive** (TP) and **False Positive** (FP) which are defined as.

- FN: variants that were present only in the ground truth but not reported by the variant caller
- TP: variants that were present in the ground truth and also reported by the variant callers, i.e., their overlap and
- FP: variants that were only reported by the variant caller but did not exist in the ground truth.

These classes were applied to all three independent analyses.

Beyond identifying TP variants, we evaluated the deviation of the callers’ AF estimates from the ground truth and thus we defined a new metric that quantifies this, as shown in Equation 3:

$$\Delta AF={AF}_{Caller}-{AF}_{GT}.$$ (3)

Further details are presented in Supplementary Material Section S8 along with the formulation of the Recall and Precision that were implemented to assess the results of the benchmark.

## 3 Results

In this section, we present the findings from our experiments based on the synthetically generated datasets in order to benchmark the variant callers. Results were structured into two sections to allow better insight. First, we provide an overview of callers’ behaviour and performance by implementing grid experiments across the genomic feature space. Second, we focus on variant classes (i.e., TP, FP, FN) from the three independent analyses: SNV true variants, SNV noise and indels.

### 3.1 Synthetic datasets

In total, 29 datasets across 620 generated files were studied. More specifically, 25 grid datasets were generated with varying coverage values (300×, 700×, 1000×, 3000× and 5000×) and different read lengths, ranging from 50 bp to 300 bp (50, 75, 100, 150 and 300 bp) to cover all the feature space, listed in Table 3.

**TABLE 3:** The 25 generated datasets for the benchmarking process. Each row represents a specified value of coverage and each column a specified read length. All datasets were named based on the convention {coverage}_{read length} (e.g., 1000_150 denotes the dataset with 1000x coverage and 150bp read length).

		Read length				
		50bp	75bp	100bp	150bp	300bp
Coverage	300x	300_50	300_75	300_100	300_150	300_300
	700x	700_50	700_75	700_100	700_150	700_300
	1000x	1000_50	1000_75	1000_100	1000_150	1000_300
	3000x	3000_50	3000_75	3000_100	3000_150	3000_300
	5000x	5000_50	5000_75	5000_100	5000_150	5000_300

The number of reads ranged on average from ∼100,000 reads, for datasets with lower coverage, to ∼300,000 reads for “deeper” datasets. Finally, four additional datasets were generated to evaluate the effect of the number of individual files on the resulting AF in the merged file, considering several scenarios (i.e., 1, 2, 4, and 10 individual files) prior to implementing the 10-file configuration described in Supplementary Material Section S12. All datasets were of high quality (i.e., properly paired reads with no secondary, duplicates, etc.) as they provide the most reliable ground truth for benchmarking.

### 3.2 Callers’ behaviour across the genomic feature space

This section presents an overview of the performance of variant callers across the genomic feature space. To systematically assess their behavior, we performed an ablation study in which individual parameters were subjected to independent variation, while all others were held constant. This approach enabled the evaluation of the influence and significance of each parameter on variant calling.

**FIGURE 4:**
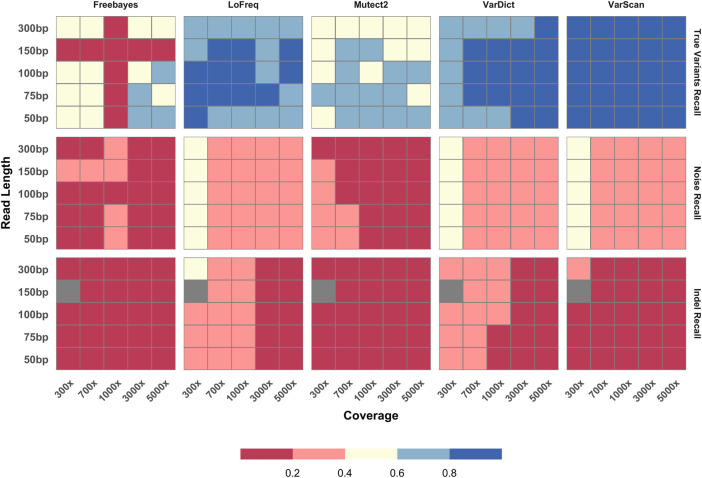
Heatmap showing Recall for all experiments across all variant callers. Rows represent read lengths and columns represent coverage values. Colors indicate Recall values, ranging from low (red) to high (blue) in increments of 0.2, with gray indicating missing values. Three independent analyses are presented: top, SNVs True Variants; middle, SNVs Noise; and bottom Indels. Each panel corresponds to a different variant caller.

We studied caller’s **FN variants trends** by studying the Recall for all grid experiments. As seen in Figure 4, Recall values were consistently higher for SNVs than indels and higher for true variants compared to noise. Among callers, VarScan2 achieved the highest Recall for true variants, but also showed the largest variability across the three independent analyses of true variants (Figure 4 top), noise (Figure 4 middle) and indels (Figure 4 bottom). VarDict and LoFreq followed, performing similarly in the noise analysis. In contrast, FreeBayes and Mutect2 yielded the lowest Recall across true variants, noise and indels.

Sequencing parameters influenced caller performance in distinct ways. In terms of noise, most callers (LoFreq, Mutect2, VarDict, VarScan2) showed better Recall at lower coverages, whereas true variant detection was more dependent on read length. Mutect2 performed best for shorter reads (50–75 bp), while LoFreq and VarDict favored the shortest (50 bp) and longest (300 bp) read lengths. All relevant data for these associations are in Supplementary Material Section S4.

**FIGURE 5:**
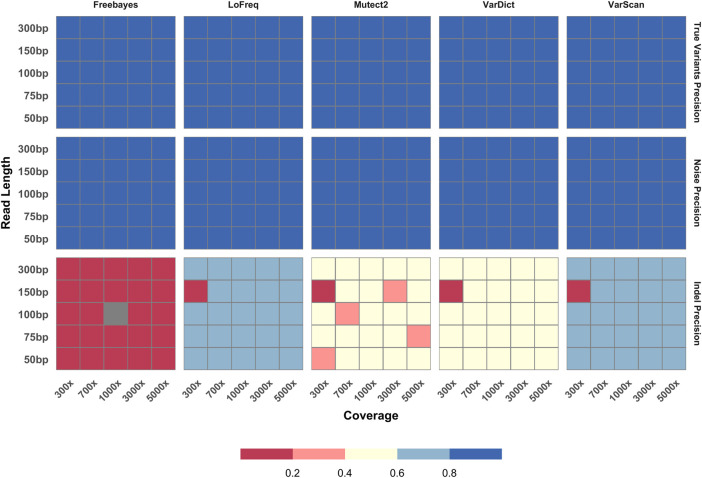
Heatmap showing Precision for all experiments across all variant callers. Rows represent varying read lengths, and columns represent coverage values. Colors indicate Precision, ranging from low (red) to high (blue) in increments of 0.2, with gray indicating missing values. Three independent analyses are presented: top, SNVs True Variants; middle, SNVs Noise; and bottom Indels. Each panel corresponds to a different variant caller.

**FP variants trends** were assessed by studying the Precision for all grid experiments. As seen in Figure 5, Precision values were consistently higher for SNVs than indels since most callers did not generate FP variants in the true variant and noise analyses. VarScan2 and Lofreq performed best in the indels analysis, followed by VarDict and Mutect2. All data for this plot are in Supplementary Material Section S4.

**FIGURE 6:**
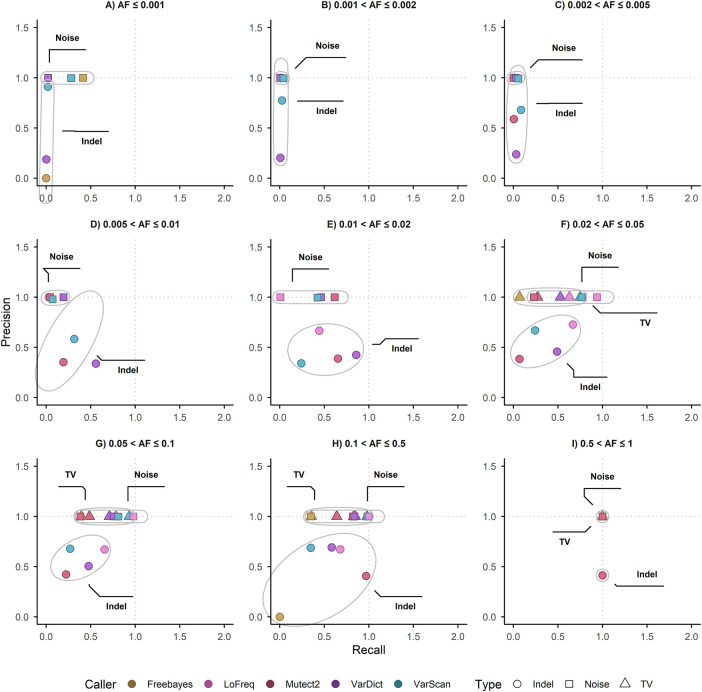
Frequency resolution of variant callers across different AF bins. Recall is plotted on the x-axis and Precision on the y-axis. Each color represents a different algorithm and each marker type corresponds to one of the three independent analyses (Indels, Noise, or True Variants [TV]). Axes are extended slightly beyond the observed metric ranges for visualization, with dashed reference lines at a value of 1 to aid interpretation. Panels correspond to AF ranges **(A)** < 0.1%, **(B)** 0.1%–0.2%, **(C)** 0.2%–0.5%, **(D)** 0.5%–1%, **(E)** 1%–2%, **(F)** 2%–5%, **(G)** 5%–10%, **(H)** 10%–50%, and **(I)** 50%–100%.

To investigate the frequency resolution of variant callers, we applied an AF binning strategy following a previous study (Xiang et al., 2023), as illustrated in Figures 6A–I. Recall and Precision metrics are shown for all callers across the different AF bins. True variants were not detected at very low frequencies (AF < 0.02, Figures 6A–E), while indels and noise were present across all frequency ranges. SNVs and indels formed separate clusters in the AF range of 0.005–0.5 (Figures 6D–H), with indels displaying a more dispersed distribution within this frequency band. Most SNV calls from most variant callers clustered tightly near high Precision (≈1.0) across all AF bins but exhibited greater variability in recall, particularly at the lowest AF values. As AF increased the points progressively concentrated toward the upper-right corner of the plot, reflecting both high Precision and high recall. Notably, indels consistently showed lower Recall and broader variability compared to SNVs and noise across all bins. Mutect2 was the only caller that reported variants from all three analyses in the higher frequency band (Figure 6I). All relevant data for this analysis are in Supplementary Material Section S5.

A further study of ΔAF was also implemented and can be found in Supplementary Material Section S9 along with all related data in Supplementary Material Section S6.

To further explore the influence of sequencing quality on caller behavior, an additional experiment introducing realistic base quality variability was performed. Preliminary results from this analysis are provided in Supplementary Material Section S10.

### 3.3 Characteristics of variant classes (TP, FP, FN)

This section analyzes how variant type influences caller performance. Results were aggregated across datasets with different coverage and read lengths to characterize overall caller behavior independent of specific sequencing settings. While earlier analyses highlight parameter-specific trends, the pooled results reveal intrinsic caller-dependent patterns in variant classes, AF distributions, and allele depth profiles. Aggregation increases statistical power and helps distinguish parameter-driven effects from consistent algorithmic tendencies, providing a generalized view of caller behavior in tumor-only settings.

**FIGURE 7:**
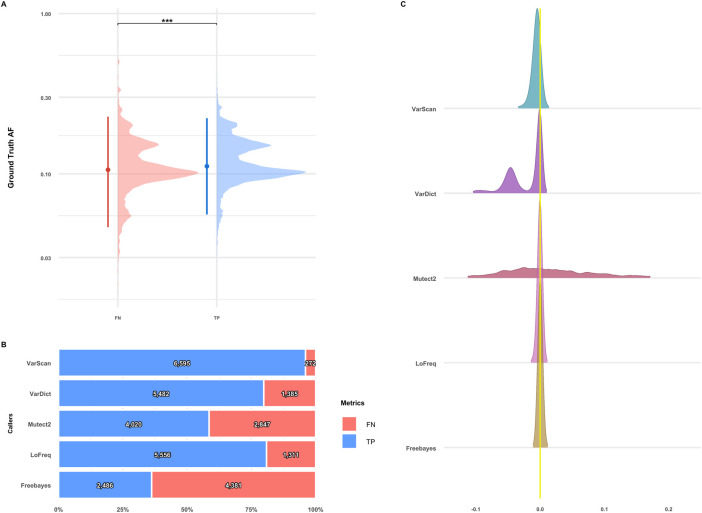
Multiplot presenting a comprehensive benchmark of variant classes (TP, FN) for the SNVs True Variants analysis. **(A)** Violin plots showing the distribution of ground truth AF for TP variants (blue) and FN variants (red). **(B)** Stacked bar charts quantifying the number of TP (blue) and FN (red) variants for all callers; bars are normalized to 100% to facilitate direct comparison of TP and FN proportions, with absolute counts displayed on each bar. **(C)** Density plots of ΔAF.

We examined SNV true variants, by aggregating results across all datasets and callers. Figure 7A shows broadly similar AF distributional shapes for FNs and TPs, while Figure 7B summarizes counts by caller. VarScan2 yielded the most TPs, followed by LoFreq and VarDict. All callers reported some FNs and no FPs, however, VarScan2 recorded the fewest (272), with LoFreq being the next best choice with 1,311 FNs. Both Mutect2 and FreeBayes showed higher numbers of FN variants. As seen in Figure 7C, which presents ΔAF densities, Mutect2 exhibited the largest dispersion around the mean. VarScan2’s density peaked at negative ΔAF and VarDict showed a secondary negative peak. By contrast, LoFreq and FreeBayes outputs remained tightly centered near zero.

**FIGURE 8:**
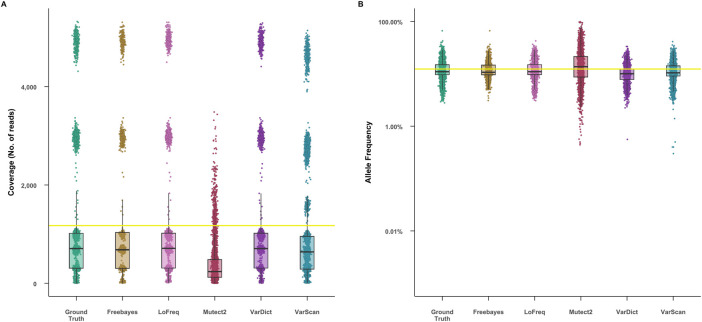
Multipanel figure illustrating the performance of all variant callers for SNVs True Variants with respect to coverage and AF compared to the ground truth. **(A)** Boxplots of coverage for all true variants in the ground truth and for each caller; each dot represents an individual variant. The yellow line indicates the mean coverage of the ground truth. **(B)** Corresponding AF boxplots for the same TP variants for each caller. The y-axis is logarithmic to better display the distribution of AF values across orders of magnitude. The yellow line indicates the mean AF in the ground truth. Each caller is shown in a distinct color, consistent across panels to facilitate direct comparison.

Diving further into true variants’ analysis, LoFreq, VarDict and VarScan2 closely “mimicked” the ground truth DP distribution, as seen in Figure 8A. Mutect2 was the outlier, with its median DP being well below the Ground Truth. A slight shift towards lower coverage values was also seen for VarScan2. Figure 8B shows that LoFreq and FreeBayes were well aligned with the Ground Truth, while VarScan2 and VarDict showed a systematic AF underestimation. Finally, Mutect2 exhibited the widest AF dispersion with its median values being above the mean of the ground truth.

**FIGURE 9:**
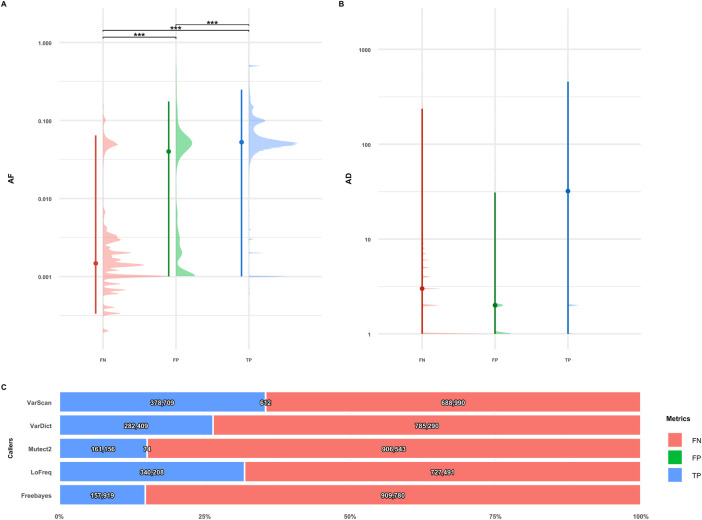
Multiplot presenting a comprehensive benchmark of variant classes (TP, FN, FP) for the SNVs Noise analysis. **(A)** Violin plots of AF for FN (red), FP (green) and TP (blue) variants across all callers; the y-axis is logarithmic. **(B)** Violin plots of alternate allele depth (AD) for FN, FP and TP variants on a logarithmic scale. **(C)** Stacked bar charts quantifying TP (blue) and FN (red) variants for all callers; bars are normalized to 100% to facilitate direct comparison of TP and FN proportions, with absolute counts displayed on each bar.

Focusing on SNV noise analysis, we aggregated results from all datasets and callers. As seen in Figure 9A, AF distributions were clearly distinguished by class with FNs being concentrated at the lowest band, FPs occupying an intermediate AF band and TPs showing the highest values. Figure 9B depicts that FNs and FPs had very low ALT depths (AD) with TPs being characterized by substantially higher AD. Class distributions were dominated by FNs across callers, as can be seen in Figure 9C. At the level of individual callers, VarScan2 reported the most TPs (∼378k) but also some FPs (612). LoFreq and VarDict reached intermediate TP efficiency (∼340k and ∼282k respectively), while Mutect2 reported few FPs (74) and a lower TP count (∼161k). Finally, FreeBayes recorded the fewest TPs (∼158k) and the highest number of FN variants (∼909k).

**FIGURE 10:**
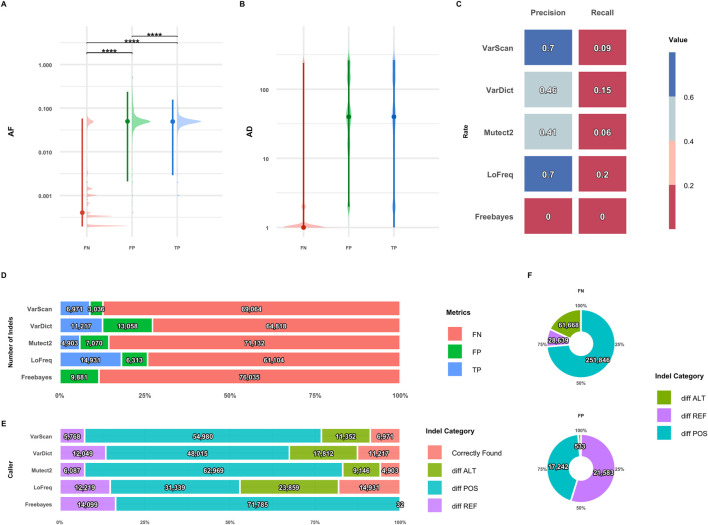
Multiplot presenting a comprehensive benchmark of variant classes (TP, FN, FP) for the indel analysis. **(A)** Violin plots of allele frequency (AF) for FN (red), FP (green) and TP (blue) variants across all callers; the y-axis is logarithmic. **(B)** Violin plots of alternate allele depth (AD) for FN, FP, and TP variants on a logarithmic scale. **(C)** Precision and Recall heatmaps for all callers. **(D)** Stacked bar charts quantifying TP (blue) and FN (red) variants for each caller; bars are normalized to 100% to allow direct comparison of proportions, with absolute counts displayed on each bar. **(E)** Stacked bar charts showing the number of indels in the categories defined in Subsection 2.2.3. **(F)** Donut charts summarizing FN (top) and FP (bottom) indel composition across the diff POS, diff REF, and diff ALT categories.

Finally, we integrated all results and data from the indel analysis. As shown in Figures 10A,B, indel FNs were concentrated at the lowest AF and AD, while FPs and TPs had the highest values with more narrow spreads. Regarding caller performance, depicted in Figures 10C,D, indel detection showed low Recall across all callers, with varying Precision; LoFreq and VarScan2 reached a Precision≈0.70 but Recall remained limited (≈0.20 and ≈0.09 respectively). VarDict attained Precision≈0.46 and Recall≈0.15, while Mutect2 showed Precision≈0.41 and Recall≈0.06. FreeBayes reported FPs without TPs (Precision = 0, Recall = 0). As seen in Figure 10D, FNs dominated every caller report, with LoFreq and VarDict yielding the largest TP totals, VarScan2 recovering fewer TPs than those two and Mutect2 and FreeBayes contributing the fewest TPs. Figure 10E offers insights regarding the indels categories, as defined in Subsection 2.2.3, showing that most mismatches arise from positional discordance (diff POS) followed by diff ALT category, across all callers. As depicted in Figure 10F, the main discordance for FN indels is diff POS, followed by diff ALT category. On the other hand, the most populated category for FP indels is that of diff REF followed by the diff POS category.

### 3.4 Runtime and computational efficiency

A runtime evaluation was conducted to assess the computational efficiency of synth4bench and the individual callers and is presented in detail in Supplementary Material Section S3. For the most demanding dataset (5000x coverage), data generation required ∼6 min. Variant calling runtimes varied substantially: Mutect2 (∼50 min), VarDict and LoFreq (∼12 min each), VarScan2 (∼3 min), and FreeBayes (∼2 min). Benchmarking and visualization required minimal additional time.

To enhance usability, we provide a concise decision table (Table 4) linking common analytical goals (e.g., low AF sensitivity, AF accuracy, indel focused workflows, conservative calling) to recommended callers and their key trade-offs. This offers an actionable summary of the benchmarking results and reinforces that caller selection should align with specific study priorities rather than a one-size-fits-all approach.

**TABLE 4:** Goal-based good practices for caller selection.

Goal	Recommended tool(s)	One-line explanation	Caveats/Limitations
Maximize sensitivity at low AF	VarScan2, VarDict	Both callers demonstrated strong recall in low-AF ranges and were among the few able to report variants below 0.1% AF.	VarScan2 shows mild AF underestimation and may introduce FPs; VarDict trades some precision for improved recall
Maximize AF estimation accuracy	LoFreq	Exhibited highly stable ΔAF distributions centered near zero across coverage and read length variations	Moderate recall sensitivity to extreme read lengths; still limited indel recall
Indel-heavy workflow (precision-focused)	LoFreq, VarScan2	Achieved the highest precision for indel detection among tested callers	Indel recall remains low across all callers; FNs dominated indel reports
Indel-heavy workflow (recall-focused)	VarDict	Recovered the largest number of TP indels and achieved the highest indel recall	Precision is lower compared to LoFreq and VarScan2
Conservative calling (minimize FPs)	FreeBayes	Produced few FP calls and showed stable AF estimates	Very low recall, particularly for indels; high FN burden
Maximize true SNV recovery	VarScan2	Reported the highest number of TP true variants across datasets	Slight negative ΔAF bias
Balanced performance across variant types	LoFreq	Demonstrated robust AF estimation, strong precision, and best overall indel performance	Recall moderately affected by extreme read lengths; indel recall still limited overall
Robustness to base-quality variation	Mutect2	Demonstrated the smallest performance changes in an exploratory analysis using modified base-quality profiles	Observation is based on a preliminary experiment

## 4 Discussion

We present synth4bench, a novel pipeline for benchmarking somatic variant callers using synthetic genomics data as ground truth. Our results show that callers differ in behavior due to their algorithmic weighting of NGS parameters. Synth4bench reveals the principles governing caller performance by analyzing diverse datasets across the NGS feature space and their responses to varying parameters.

Synth4bench offers a unique combination of benchmarking-oriented features that extend beyond synthetic read generation alone. First, the framework provides additional granularity for indel evaluation by systematically examining indel mismatch behavior through dedicated categorization strategies. Furthermore, all evaluated variant callers and associated software tools were incorporated into a unified Conda environment, substantially improving usability and reproducibility by minimizing dependency conflicts and complex installation procedures commonly encountered in variant calling workflows. In addition, a series of harmonization steps were implemented to enable more consistent comparison across callers and reporting. Also, the complete step-by-step bioinformatics pipeline, together with all relevant commands presented in detail in Table 1, represents an additional strength of the framework by improving transparency for future benchmarking studies and downstream methodological comparisons. Another key characteristic of synth4bench is the implementation of three independent analyses for true variants, noise and indels, enabling more transparent evaluation of caller-specific behavior while explicitly accounting for background noise. Furthermore, in support of open science and reuse by the wider community, all generated datasets and associated developed software are publicly available through openly accessible repositories. Finally, rather than relying on randomly introduced spike-in variants, synth4bench follows a controlled read dilution methodology, enabling systematic benchmarking of low-frequency somatic variant detection under predefined sequencing conditions.

Recent studies investigating **low-frequency variant detection** have frequently classified AF values ≤ 10% as low-frequency, values below 5% as very low-frequency and variants ≤1% AF as extremely low-frequency (Xiang et al., 2023; Ha et al., 2023; Sergi et al., 2024; Chen et al., 2020). In alignment with this classification, our analysis used 10% AF as the primary benchmarking threshold, however, the generated datasets also included variants extending to lower frequencies, reaching approximately 2% AF for true variants (Figure 6F) and as low as 0.1% AF within the indel and noise analyses (Figures 6A–E).

We mainly focused on the **technical evaluation** of variant callers, hence variant annotation was not incorporated. While biological context, such as certain genomic regions or hotspots, is undoubtedly important, our approach deliberately isolated methodological performance, allowing the observed differences between callers to reflect technical factors.

**Read alignment** was deliberately excluded because NEAT produces aligned reads, allowing us to isolate variant caller performance from mapping-related variability. The goal was to evaluate caller-specific behavior under fully controlled conditions, focusing strictly on variant detection. While mapping errors are relevant in real-world workflows, including them would introduce additional confounding factors and obscure methodological differences between callers. Synth4bench therefore evaluates caller performance in isolation, with alignment effects intentionally outside the scope of this technical benchmark. However, this process step will also be incorporated in our pipeline.

Comparison of our results with those from recent benchmarking studies revealed several consistent observations. In specific, the limited inter-caller agreement observed in our analyses is consistent with previous findings reporting substantial variability among variant callers, particularly for low-frequency variants and challenging sequencing conditions (He et al., 2021). This disagreement was even more pronounced for indels, where callers frequently differed in both detection and variant representation, further highlighting the increased complexity of indel calling strategies (Guille et al., 2024; He et al., 2021). In further agreement with recent studies on synthetic data, our analyses also showed that indel detection generally resulted in lower precision values compared to SNV detection, even among the better-performing callers (Sergi et al., 2024). Furthermore, we observed substantial differences between caller-specific detection limits, reinforcing previous reports demonstrating the heterogeneous sensitivity profiles of currently available somatic variant callers (He et al., 2021).

Our analysis revealed distinct patterns in **Recall and Precision** performance across variant callers, variant types and NGS configurations. The higher Recall for SNVs compared to indels and for true variants compared to noise, is consistent with known challenges in indel detection and highlights the greater reliability of callers for SNVs (Guille et al., 2024; Ha et al., 2023; Kumaran et al., 2019; Xu, 2018; Sandmann et al., 2018; Hasan et al., 2015; Ghoneim et al., 2014). The dependence of Recall on NGS parameters further emphasizes the interplay between algorithmic design and data characteristics. As also shown by a recent benchmarking study (Xiang et al., 2023), increasing sequencing depth does not necessarily lead to uniform performance improvement across all variant callers, as different algorithms respond differently to increasing read depth and background signal. Consistent with these observations, declining Recall trends with increasing coverage were observed in our noise analyses for LoFreq, Mutect2, VarDict and VarScan2, as well as in the indel analyses for LoFreq and VarDict, where accurate variant discrimination is inherently more challenging. Furthermore, examination of the FN distributions in Figures 9A, 10A showed that the majority of missed variants were concentrated at very low AF values. Therefore, as sequencing coverage increased, additional low-frequency background signal and noise were also introduced into the datasets, potentially increasing the difficulty of distinguishing true low-frequency variants from artifacts.

It was observed that read length had a more decisive effect on true variant detection (Figure 4). The absence of FP for true variants and noise (Figure 5) can be largely attributed to the way these analyses were constructed. By defining all non-true variants as noise, the pipeline effectively eliminated scenarios where FP could occur. This design highlights the limitations of interpreting absolute Precision values in isolation, as they may reflect dataset composition rather than caller performance alone.

The joint evaluation of Recall and Precision across AF bins (Figure 6) highlights callers’ **AF resolution**. In the intermediate range (0.005–0.5) (Figures 6D–H), SNVs and indels became more distinct; SNVs clustered tightly, while indels remained dispersed, consistent with their known detection difficulties (Guille et al., 2024; Ha et al., 2023; Kumaran et al., 2019; Xu, 2018; Sandmann et al., 2018; Hasan et al., 2015; Ghoneim et al., 2014). This separation indicates that indel calling is more error-prone and AF-sensitive, reflecting algorithmic limitations at low frequencies. Overall, higher AF stabilized performance and improved recall. Callers such as VarScan2 and VarDict appear better suited for detecting low-AF variants.

For **indels analysis**, Recall remained uniformly low across callers, even when Precision was moderate to high, driven by FNs at very low AF (Figure 10C). Our analysis adds granularity to indel mismatches; FN indels mainly resulted from chromosomal positional errors (diff POS), whereas FP indels stemmed from both reference mismatches (diff REF) and positional inaccuracies (diff POS) (Figure 10F). Caller selection should align with study aims, LoFreq and VarScan2 favor Precision-focused workflows, while VarDict best supports Recall-oriented analyses, offering the highest Recall with lower trade-off.

Regarding **true variants**, our results indicate that adequate candidates for their detection are VarScan2 for maximizing TP recovery (Figure 7B), but with awareness of its mild AF underestimation (Figure 7C) and its tendency to produce FP variants, and LoFreq when precise AF estimation is paramount (Figure 7C).

The performance of **Freebayes** in our somatic variant benchmark was notably constrained, yielding the poorest results. This outcome is consistent with the fact that Freebayes was not initially developed for somatic variant calling, instead employing a Bayesian, haplotype-based framework that models genotype likelihoods and infers ploidy across a cohort (Garrison and Marth, 2012; Hollizeck et al., 2021). Although FreeBayes was not specifically designed for tumor-only somatic calling, it was included as a representative haplotype-based Bayesian caller and as a comparative baseline. Selection criteria prioritized widely used, open-source tools capable of detecting both SNVs and indels, regardless of their original optimization context. Including FreeBayes broadens the methodological spectrum and highlights how algorithmic design influences performance under tumor-only conditions, serving as an informative reference rather than a critique of its intended use. The primary source of discordance was systematic misrepresentation of the REF allele, especially for indels. Notably, FreeBayes produced no TP indels (Figure 10D) and underperformed yielding low Recall (Figure 4). Despite limited sensitivity, it showed stable AF estimation precision, with a low ΔAF standard deviation (Figure 7C). However, ΔAF extensive analyses across read length and coverage (Supplementary Figure S1), revealed mild biases, suggesting reduced robustness compared to LoFreq and VarDict. Overall, the combination of high Precision but numerous FNs indicates that FreeBayes is conservative, favoring fewer, more accurate calls at the cost of Recall.

**Mutect2**’s performance showed a complex pattern, partly consistent with prior reports. Although some studies highlight its strength in detecting low-AF SNVs (Cai et al., 2016), our results revealed consistently low Recall (Figure 4), indicating limited sensitivity. Mutect2 frames somatic detection as a Bayesian hypothesis test on allele fraction, running two models (reference $M_{0}$ and variant $M_{m}^{f}$ ) to distinguish true variants from sequencing errors (Cibulskis et al., 2013), assuming independent base errors like LoFreq. A key challenge noted in prior work is Mutect2’s tendency to produce a substantial number of FP calls in its tumor-only mode (Ha et al., 2023), a common issue for methods that rely on filtering germline mutations using population databases (Jones et al., 2015). Regarding ΔAF, Mutect2 performed better with short reads but was vulnerable to longer reads and higher coverage (Supplementary Figure S1). For indels, it was notably conservative, exhibiting low Precision across the entire AF spectrum (Figure 6). While its Recall increased as AF reached higher values, its overall sensitivity remained constrained. Furthermore, its AF estimates were the noisiest (Figure 7C), largely due to internal read downsampling (Figures 8A,B), which reduced effective coverage and widened AF dispersion, ultimately lowering recall, especially for low-AF variants even at high ground truth coverage.

**VarScan2** differs from haplotype-based callers by using pileup input, enabling full position visibility before applying heuristic filters and thresholds (Koboldt et al., 2012). This approach yielded mixed, yet generally robust results in our benchmark. VarScan2 demonstrated remarkable capabilities in detecting both indels and low-frequency SNVs, being one of the few callers able to report variants with an AF below 0.1% (Figures 6A–D). Its strong overall Recall (Figure 4) suggests it can be a highly effective tool for true variant detection. For indels, it exhibited higher Precision than some other callers but still missed a significant number of TP indels (Figure 10), indicating a limitation in its sensitivity for this variant type. Regarding AF estimation, VarScan2’s performance was generally good (Supplementary Figure S1 and Figure 7C). However, its ΔAF distributions often showed a slight negative-centered bias (Figure 7C), indicating a tendency to underestimate AF. This suggests that while it is robust, it may be less stable or accurate than algorithms like LoFreq or VarDict under varying read length and coverage conditions (Figure 5). A particularly noteworthy finding came from the noise analysis (Figure 9C), where VarScan2 successfully captured the most TP noise variants compared to all other algorithms. This further underscores its sensitivity, making it suitable for identifying true variants.

The performance of **LoFreq** in our benchmark demonstrated exceptional robustness and Precision, aligning with its established strengths in detecting rare variants (Wilm et al., 2012) and its capacity to discern TP reported in previous studies (Xiang et al., 2023). LoFreq’s core methodology is rooted in a null hypothesis that assumes all variants arise from sequencing error. It models the presence of a variant as a series of independent Bernoulli trials, with each base’s error probability directly informed by its Phred quality score. This heavy reliance on quality scores makes our well-characterized benchmark an ideal scenario for studying LoFreq. This methodology, however, represents both a strength and a potential weakness, as it introduces an inherent bias (Wilm et al., 2012). Despite this, LoFreq’s practical performance was highly favorable. Its ability to call rare variants was confirmed, and its consistent accuracy and precision in AF estimation across the entire feature space (Supplementary Figure S1) demonstrated remarkable robustness against variations in read length and sequencing coverage. Concerning indels, LoFreq was the best-performing caller, yielding the largest TP totals, while also achieving the highest Precision (Figure 10). Although it still missed some TP indels, its overall performance in this challenging category was superior. For overall Recall (Figure 4) LoFreq was moderately affected by the read length, with a slight Recall decrease at extreme lengths (50 bp and 300 bp). Finally, in the noise analysis (Figure 9C), LoFreq was the second most successful algorithm, after VarScan2, at capturing TP noise variants. While we did not observe the large numbers of FP reported in some prior studies (Olson et al., 2023; Xiang et al., 2023; Ha et al., 2023), likely a result of our data generation methodology taking as noise all low-AF variants that were excluded from the true variant analysis as explained in Section 2.2.2, LoFreq’s high Precision suggests it effectively manages to detect low-frequency variants. This overall profile establishes LoFreq as one of the most reliable and technology-independent callers (Wilm et al., 2012) for robust AF estimation across diverse sequencing conditions.

**VarDict** implements a robust strategy for variant calling that significantly improves indel sensitivity by performing local re-alignment. As detailed in previous work (Lai et al., 2016), VarDict leverages read information by treating mismatches and soft-clips as potential indel clues. By locally re-aligning read ends (a supervised approach) and mining shared soft-clip sites that flank potential indel regions (unsupervised), it effectively recovers hidden evidence and reports more accurate AFs. This methodological approach contributed to VarDict’s strong performance in AF estimation. During the study of ΔAF (Supplementary Figure S1 and Figure 7C), VarDict demonstrated exceptional robustness and reliability, exhibiting highly consistent accuracy and precision across all genomic feature space. However, a slight tendency to underestimate AF was noted, with its ΔAF distributions showing a second peak at negative values indicating a bias (Figure 7C). For overall Recall (Figure 4) similarly to LoFreq, its performance was also moderately affected by the read length, with a slight Recall decrease at extreme lengths (50 bp and 300 bp). VarDict’s local re-alignment strategy was particularly impactful for indels. It ranked second for TP indel recovery and achieved the highest overall indel Recall (albeit a modest ∼0.15) by effectively minimizing FN.

Regarding runtime and computational efficiency, our results highlight that computational cost differs markedly across tools and should be considered alongside accuracy when selecting a caller.

Our overall results are consistent with prior reports showing **persistent disagreement** among variant callers, with minimal overlap even on identical samples (Cai et al., 2016; Sandmann et al., 2018; Roberts et al., 2013). Moreover, benchmarking studies themselves yield inconsistent outcomes, reflecting strong dependence on input data, a limitation that persists even in ML-based ensemble methods (Sandmann et al., 2018; Wang et al., 2020). These inconsistencies suggest that current algorithms still fail to fully capture mutational mechanisms and NGS artifacts, indicating that fully modeling of the underlying biological and technical processes remains an open challenge.

All analyses were performed on the raw outputs of each caller without the application of caller-specific post-calling filters, as the objective was to evaluate intrinsic caller behavior.

Although the present technical benchmark was extensively evaluated within a **locus-specific setting**, the modular and fully parameterizable design of the pipeline supports its potential adaptation and implementation across additional gene loci and potentially even in broader genome-wide benchmarking settings, with only minor modifications to the workflow. In its current form, the workflow was implemented as an exemplary proof-of-concept focused on a specific genomic region for computational efficiency and controlled benchmarking.

An additional exploratory analysis was performed to investigate the influence of realistic **base quality** variability on variant caller performance. Although these are only preliminary results, aimed to highlight the versatility of synth4bench it demonstrated that callers responded differently to modified base quality profiles. Among the evaluated tools, Mutect2 exhibited the smallest changes in TP, FP and FN classifications, suggesting stable performance under the tested quality perturbation. In contrast, other callers displayed more pronounced shifts in their performance. These caller-specific responses further highlight that variant callers differ not only in their overall performance characteristics but also in how sequencing quality information is incorporated into their underlying decision-making processes. Consequently, changes in base-quality distributions may influence callers in distinct ways, reflecting the different underlying assumptions employed by each algorithm. These observations indicate that robustness to sequencing quality variation may represent an additional practical consideration when selecting a variant caller, particularly for studies involving archived samples or sequencing datasets with variable quality. As this analysis was preliminary, further investigation across additional quality profiles and real-world sequencing datasets is warranted before generalizing these observations.

Although **ensemble approaches** were not evaluated in this study, synth4bench provides a structured foundation for their development and validation. By generating controlled ground truth datasets and systematically quantifying Recall, Precision, ΔAF, and variant-class composition, the pipeline enables reproducible evaluation of complementary caller characteristics. For instance, VarDict shows higher Recall (especially for indels), while LoFreq demonstrates strong Precision and stable AF estimation. An ensemble combining such strengths could better balance sensitivity and specificity. Synth4bench can therefore serve as a platform for training and validating consensus or machine learning–based meta-callers to address the observed performance trade-offs.

Real-world benchmarking datasets capture important biological variability and technical artifacts arising from sequencing and downstream analysis workflows. However, their increased complexity can also introduce uncertainty during the process of benchmarking, particularly due to incomplete characterization of the underlying ground truth, a challenge that may persist even in highly curated cell-line-based datasets. In contrast, synthetic benchmarking frameworks provide controlled experimental conditions and explicitly defined ground truth, enabling systematic evaluation of caller behavior under predefined sequencing and variant configurations. As recently noted in (Guille et al., 2024), many existing benchmarking approaches remain constrained by the availability and characteristics of suitable biological samples, particularly for deep targeted sequencing and low-frequency somatic variant detection. Nevertheless, synthetic datasets should not be viewed as a replacement for real-world biological data, but rather as a complementary method for controlled technical benchmarking, while the ultimate objective remains the validation of computational methodologies against real biological datasets. To further explore this relationship, an additional preliminary analysis using variants from two independent GIAB benchmark samples (HG001 and HG002) was performed within the synth4bench framework as a brief assessment. Consistent caller-specific recovery trends were observed across these biologically derived benchmark variants (Supplementary Section 11).

## 5 Conclusion

In summary, this work provides a systematic technical benchmark of widely used variant calling algorithms, focusing on their ability to detect both SNVs and indels under controlled, high-quality conditions as offered by the generation of synthetic data. Our findings highlight substantial **discrepancies** among callers, consistent with previous reports and reinforce the notion that existing methods do not yet fully capture the complexity of mutational mechanisms, leaving this an important open challenge for the field. **Indels** remain the hardest variant type to call, and tool selection must be tailored, favoring LoFreq or VarScan2 for Precision-focused indel calling or VarDict when Recall is prioritized. The trade-off between Recall and Precision is most acute at low AF, with VarScan2 and VarDict demonstrating suitability for variant detection at this challenging lower end of the AF **spectrum**. For **true SNV variants**, VarScan2 is optimal for maximizing TP recovery, while LoFreq is recommended when precise AF estimation is paramount. Ultimately, achieving maximal **sensitivity and reliability** necessitates caller-specific optimization of sequencing strategies, with an informed choice of algorithm based directly on the study’s priorities.

Despite all efforts, this systematic benchmark has potential pitfalls. We deliberately restricted our analyses to high-quality synthetic datasets in order to establish a controlled and reliable ground truth for benchmarking caller performance. By excluding additional biological and technical artifacts, we aimed to isolate algorithmic behavior and avoid confounding factors, since inherent noise and inconsistencies from lower-quality or noisy data could obscure genuine differences between variant calling methods. Although real sequencing data involve complex sources of variability, modeling all such effects was beyond the scope of this technical benchmark. Our objective was therefore not to replicate full biological complexity, but to evaluate variant callers under fully defined and reproducible conditions, while acknowledging that validation on real-world data remains essential. Disagreement observed even in such controlled settings highlights fundamental algorithmic differences that are likely to be further amplified in real-world datasets characterized by biological variability and technical artifacts. Therefore, the pipeline should be interpreted as a technical stress test of caller behavior rather than an attempt to emulate clinical sequencing conditions. Furthermore, there is the aspect of the quality of synthetic data itself. There are various evaluation metrics with respect to fidelity, privacy and applicability, which have been well-addressed by an organized effort by the ELIXIR AI Ecosystem Focus Group (Fragkouli et al., 2026). Finally, this work focuses primarily on variant calling itself not including read alignment, since a previous study (Kumaran et al., 2019) demonstrated that the choice of variant caller exerts a greater influence on the detection of SNVs and indels than the choice of alignment algorithm. We acknowledge that using simulator-generated aligned reads excludes mapping-related errors present in real workflows. This was intentional, as our goal was to isolate the intrinsic behavior of variant callers under controlled conditions. By removing alignment variability, we ensured that differences in Recall, Precision, ΔAF and indel classification reflect methodological characteristics rather than compound pipeline effects. Our conclusions should therefore be interpreted as a technical benchmark of caller performance in isolation, not as a full end-to-end clinical workflow simulation.

Given the astronomical number of potential parameter combinations, as already noted by (Sandmann et al., 2017), and fully exploring the entire parameter space for every algorithm was computationally prohibitive and beyond the scope of this work. Therefore, our study necessarily focused on a fixed set of parameters for each variant caller, selected according to their best practices. We noted that none of the callers proposed specific instructions for a tumor-only mode. Also, this study focused on two key NGS parameters, sequencing depth and read length, to create a structured and computationally manageable benchmarking pipeline. Although additional factors such as fragment length, base quality, error calibration, GC bias, duplicates and repetitive regions can affect performance, exploring all combinations was beyond the scope of this work. We therefore prioritized controlled variation of selected parameters to isolate algorithmic behavior, with future extensions able to incorporate additional sequencing characteristics to better approximate real-world complexity.

Finally, while synthetic data are highly valuable for developing and benchmarking tools and easy to generate for modeling extreme or impossible wet-lab scenarios, it is merely a means to an end. The ultimate objective remains evaluating hypotheses against real data.

## Data Availability

Aligning with open science practices (Farrell et al., 2026), all necessary resources required for the execution of synth4bench are available and ready to use. Code: https://github.com/sfragkoul/synth4bench/ Release: https://zenodo.org/records/19049171 All synthetic datasets are openly available in Zenodo (https://zenodo.org/records/16524193).
