# Supplementary material for: Synth4bench: generating synthetic data for benchmarking tumor-only somatic variant calling algorithms
Source: Front Bioinform. 2026 Jul 16;6:1858375. doi: 10.3389/fbinf.2026.1858375 (PMC13422537; doi:10.3389/fbinf.2026.1858375)
Supplement: Supplementary file 1 [file DataSheet1.pdf]

Supplementary Material

S1 NEAT parameters

A subset of NEAT input parameters that can be set by the user, their corresponding description taken from the documentation and the used range for this work.

Parameter	Description	Value
coverage	Average coverage	300-5000
read length	The desired read length	50-300
avg mut rate	Rescale avg mutation rate to this (1/bp)	0.1
ploidy	Desired ploidy	2
rng	rng seed value	varying
pe	Paired-end fragment length mean and std	(300, 30)

S2 Synthetic data generation seeds

Seed	File Name	Command Arguments
1	700_100_7/1	-M 0.1 -R 150 -c 100 --pe 300 30 --bam --vcf
2	700_100_7/2	-M 0.1 -R 150 -c 100 --pe 300 30 --bam --vcf
3	700_100_7/3	-M 0.1 -R 150 -c 100 --pe 300 30 --bam --vcf
4	700_100_7/4	-M 0.1 -R 150 -c 100 --pe 300 30 --bam --vcf
5	700_100_7/5	-M 0.1 -R 150 -c 100 --pe 300 30 --bam --vcf
16	700_100_7/6	-M 0.1 -R 150 -c 100 --pe 300 30 --bam --vcf
7	700_100_7/7	-M 0.1 -R 150 -c 100 --pe 300 30 --bam --vcf
8	700_70_10/1	-M 0.1 -R 150 -c 70 --pe 300 30 --bam --vcf
9	700_70_10/2	-M 0.1 -R 150 -c 70 --pe 300 30 --bam --vcf
10	700_70_10/3	-M 0.1 -R 150 -c 70 --pe 300 30 --bam --vcf
12	700_70_10/4	-M 0.1 -R 150 -c 70 --pe 300 30 --bam --vcf
13	700_70_10/5	-M 0.1 -R 150 -c 70 --pe 300 30 --bam --vcf
14	700_70_10/6	-M 0.1 -R 150 -c 70 --pe 300 30 --bam --vcf
15	700_70_10/7	-M 0.1 -R 150 -c 70 --pe 300 30 --bam --vcf
17	700_70_10/8	-M 0.1 -R 150 -c 70 --pe 300 30 --bam --vcf
19	700_70_10/9	-M 0.1 -R 150 -c 70 --pe 300 30 --bam --vcf
21	700_70_10/10	-M 0.1 -R 150 -c 70 --pe 300 30 --bam --vcf
22	1000_1000_1	-M 0.1 -R 150 -c 1000 --pe 300 30 --bam --vcf
23	1000_500_2/1	-M 0.1 -R 150 -c 500 --pe 300 30 --bam --vcf
29	1000_500_2/2	-M 0.1 -R 150 -c 500 --pe 300 30 --bam --vcf
30	1000_250_4/1	-M 0.1 -R 150 -c 250 --pe 300 30 --bam --vcf
33	1000_250_4/2	-M 0.1 -R 150 -c 250 --pe 300 30 --bam --vcf
34	1000_250_4/3	-M 0.1 -R 150 -c 250 --pe 300 30 --bam --vcf
37	1000_250_4/4	-M 0.1 -R 150 -c 250 --pe 300 30 --bam --vcf
38	1000_100_10/1	-M 0.1 -R 150 -c 100 --pe 300 30 --bam --vcf
43	1000_100_10/2	-M 0.1 -R 150 -c 100 --pe 300 30 --bam --vcf
44	1000_100_10/3	-M 0.1 -R 150 -c 100 --pe 300 30 --bam --vcf
49	1000_100_10/4	-M 0.1 -R 150 -c 100 --pe 300 30 --bam --vcf
51	1000_100_10/5	-M 0.1 -R 150 -c 100 --pe 300 30 --bam --vcf
52	1000_100_10/6	-M 0.1 -R 150 -c 100 --pe 300 30 --bam --vcf

53	1000_100_10/7	-M 0.1 -R 150 -c 100 --pe 300 30 --bam --vcf
54	1000_100_10/8	-M 0.1 -R 150 -c 100 --pe 300 30 --bam --vcf
57	1000_100_10/9	-M 0.1 -R 150 -c 100 --pe 300 30 --bam --vcf
58	1000_100_10/10	-M 0.1 -R 150 -c 100 --pe 300 30 --bam --vcf
60	300_30_10/1	-M 0.1 -R 150 -c 30 --pe 300 30 --bam --vcf
61	300_30_10/2	-M 0.1 -R 150 -c 30 --pe 300 30 --bam --vcf
62	300_30_10/3	-M 0.1 -R 150 -c 30 --pe 300 30 --bam --vcf
65	300_30_10/4	-M 0.1 -R 150 -c 30 --pe 300 30 --bam --vcf
66	300_30_10/5	-M 0.1 -R 150 -c 30 --pe 300 30 --bam --vcf
67	300_30_10/6	-M 0.1 -R 150 -c 30 --pe 300 30 --bam --vcf
70	300_30_10/7	-M 0.1 -R 150 -c 30 --pe 300 30 --bam --vcf
71	300_30_10/8	-M 0.1 -R 150 -c 30 --pe 300 30 --bam --vcf
73	300_30_10/9	-M 0.1 -R 150 -c 30 --pe 300 30 --bam --vcf
76	300_30_10/10	-M 0.1 -R 150 -c 30 --pe 300 30 --bam --vcf
78	3000_300_10/1	-M 0.1 -R 150 -c 300 --pe 300 30 --bam --vcf
79	3000_300_10/2	-M 0.1 -R 150 -c 300 --pe 300 30 --bam --vcf
80	3000_300_10/3	-M 0.1 -R 150 -c 300 --pe 300 30 --bam --vcf
81	3000_300_10/4	-M 0.1 -R 150 -c 300 --pe 300 30 --bam --vcf
83	3000_300_10/5	-M 0.1 -R 150 -c 300 --pe 300 30 --bam --vcf
85	3000_300_10/6	-M 0.1 -R 150 -c 300 --pe 300 30 --bam --vcf
87	3000_300_10/7	-M 0.1 -R 150 -c 300 --pe 300 30 --bam --vcf
91	3000_300_10/8	-M 0.1 -R 150 -c 300 --pe 300 30 --bam --vcf
92	3000_300_10/9	-M 0.1 -R 150 -c 300 --pe 300 30 --bam --vcf
93	3000_300_10/10	-M 0.1 -R 150 -c 300 --pe 300 30 --bam --vcf
95	5000_500_10/1	-M 0.1 -R 150 -c 500 --pe 300 30 --bam --vcf
96	5000_500_10/2	-M 0.1 -R 150 -c 500 --pe 300 30 --bam --vcf
97	5000_500_10/3	-M 0.1 -R 150 -c 500 --pe 300 30 --bam --vcf
98	5000_500_10/4	-M 0.1 -R 150 -c 500 --pe 300 30 --bam --vcf
100	5000_500_10/5	-M 0.1 -R 150 -c 500 --pe 300 30 --bam --vcf
102	5000_500_10/6	-M 0.1 -R 150 -c 500 --pe 300 30 --bam --vcf
104	5000_500_10/7	-M 0.1 -R 150 -c 500 --pe 300 30 --bam --vcf
105	5000_500_10/8	-M 0.1 -R 150 -c 500 --pe 300 30 --bam --vcf

106	5000_500_10/9	-M 0.1 -R 150 -c 500 --pe 300 30 --bam --vcf
110	5000_500_10/10	-M 0.1 -R 150 -c 500 --pe 300 30 --bam --vcf
113	1000_75/1	-M 0.1 -R 75 -c 100 --pe 300 30 --bam --vcf
116	1000_75/2	-M 0.1 -R 75 -c 100 --pe 300 30 --bam --vcf
117	1000_75/3	-M 0.1 -R 75 -c 100 --pe 300 30 --bam --vcf
118	1000_75/4	-M 0.1 -R 75 -c 100 --pe 300 30 --bam --vcf
120	1000_75/5	-M 0.1 -R 75 -c 100 --pe 300 30 --bam --vcf
121	1000_75/6	-M 0.1 -R 75 -c 100 --pe 300 30 --bam --vcf
123	1000_75/7	-M 0.1 -R 75 -c 100 --pe 300 30 --bam --vcf
125	1000_75/8	-M 0.1 -R 75 -c 100 --pe 300 30 --bam --vcf
129	1000_75/9	-M 0.1 -R 75 -c 100 --pe 300 30 --bam --vcf
130	1000_75/10	-M 0.1 -R 75 -c 100 --pe 300 30 --bam --vcf
131	1000_300/1	-M 0.1 -R 300 -c 100 --pe 300 30 --bam --vcf
133	1000_300/2	-M 0.1 -R 300 -c 100 --pe 300 30 --bam --vcf
134	1000_300/3	-M 0.1 -R 300 -c 100 --pe 300 30 --bam --vcf
135	1000_300/4	-M 0.1 -R 300 -c 100 --pe 300 30 --bam --vcf
138	1000_300/5	-M 0.1 -R 300 -c 100 --pe 300 30 --bam --vcf
141	1000_300/6	-M 0.1 -R 300 -c 100 --pe 300 30 --bam --vcf
143	1000_300/7	-M 0.1 -R 300 -c 100 --pe 300 30 --bam --vcf
147	1000_300/8	-M 0.1 -R 300 -c 100 --pe 300 30 --bam --vcf
149	1000_300/9	-M 0.1 -R 300 -c 100 --pe 300 30 --bam --vcf
151	1000_300/10	-M 0.1 -R 300 -c 100 --pe 300 30 --bam --vcf
154	1000_50/1	-M 0.1 -R 50 -c 100 --pe 300 30 --bam --vcf
156	1000_50/2	-M 0.1 -R 50 -c 100 --pe 300 30 --bam --vcf
157	1000_50/3	-M 0.1 -R 50 -c 100 --pe 300 30 --bam --vcf
158	1000_50/4	-M 0.1 -R 50 -c 100 --pe 300 30 --bam --vcf
159	1000_50/5	-M 0.1 -R 50 -c 100 --pe 300 30 --bam --vcf
162	1000_50/6	-M 0.1 -R 50 -c 100 --pe 300 30 --bam --vcf
163	1000_50/7	-M 0.1 -R 50 -c 100 --pe 300 30 --bam --vcf
164	1000_50/8	-M 0.1 -R 50 -c 100 --pe 300 30 --bam --vcf
166	1000_50/9	-M 0.1 -R 50 -c 100 --pe 300 30 --bam --vcf
169	1000_50/10	-M 0.1 -R 50 -c 100 --pe 300 30 --bam --vcf

170	1000_170/1	-M 0.1 -R 170 -c 100 --pe 300 30 --bam --vcf
171	1000_170/2	-M 0.1 -R 170 -c 100 --pe 300 30 --bam --vcf
172	1000_170/3	-M 0.1 -R 170 -c 100 --pe 300 30 --bam --vcf
174	1000_170/4	-M 0.1 -R 170 -c 100 --pe 300 30 --bam --vcf
182	1000_170/5	-M 0.1 -R 170 -c 100 --pe 300 30 --bam --vcf
183	1000_170/6	-M 0.1 -R 170 -c 100 --pe 300 30 --bam --vcf
185	1000_170/7	-M 0.1 -R 170 -c 100 --pe 300 30 --bam --vcf
186	1000_170/8	-M 0.1 -R 170 -c 100 --pe 300 30 --bam --vcf
187	1000_170/9	-M 0.1 -R 170 -c 100 --pe 300 30 --bam --vcf
188	1000_170/10	-M 0.1 -R 170 -c 100 --pe 300 30 --bam --vcf
189	1000_25/1	-M 0.1 -R 25 -c 100 --pe 300 30 --bam --vcf
193	1000_25/2	-M 0.1 -R 25 -c 100 --pe 300 30 --bam --vcf
194	1000_25/3	-M 0.1 -R 25 -c 100 --pe 300 30 --bam --vcf
198	1000_25/4	-M 0.1 -R 25 -c 100 --pe 300 30 --bam --vcf
200	1000_25/5	-M 0.1 -R 25 -c 100 --pe 300 30 --bam --vcf
201	1000_25/6	-M 0.1 -R 25 -c 100 --pe 300 30 --bam --vcf
205	1000_25/7	-M 0.1 -R 25 -c 100 --pe 300 30 --bam --vcf
206	1000_25/8	-M 0.1 -R 25 -c 100 --pe 300 30 --bam --vcf
207	1000_25/9	-M 0.1 -R 25 -c 100 --pe 300 30 --bam --vcf
211	1000_25/10	-M 0.1 -R 25 -c 100 --pe 300 30 --bam --vcf
213	1000_100/1	-M 0.1 -R 100 -c 100 --pe 300 30 --bam --vcf
214	1000_100/2	-M 0.1 -R 100 -c 100 --pe 300 30 --bam --vcf
215	1000_100/3	-M 0.1 -R 100 -c 100 --pe 300 30 --bam --vcf
217	1000_100/4	-M 0.1 -R 100 -c 100 --pe 300 30 --bam --vcf
218	1000_100/5	-M 0.1 -R 100 -c 100 --pe 300 30 --bam --vcf
225	1000_100/6	-M 0.1 -R 100 -c 100 --pe 300 30 --bam --vcf
226	1000_100/7	-M 0.1 -R 100 -c 100 --pe 300 30 --bam --vcf
228	1000_100/8	-M 0.1 -R 100 -c 100 --pe 300 30 --bam --vcf
229	1000_100/9	-M 0.1 -R 100 -c 100 --pe 300 30 --bam --vcf
230	1000_100/10	-M 0.1 -R 100 -c 100 --pe 300 30 --bam --vcf
232	1000_200/1	-M 0.1 -R 200 -c 100 --pe 300 30 --bam --vcf
234	1000_200/2	-M 0.1 -R 200 -c 100 --pe 300 30 --bam --vcf

236	1000_200/3	-M 0.1 -R 200 -c 100 --pe 300 30 --bam --vcf
238	1000_200/4	-M 0.1 -R 200 -c 100 --pe 300 30 --bam --vcf
241	1000_200/5	-M 0.1 -R 200 -c 100 --pe 300 30 --bam --vcf
248	1000_200/6	-M 0.1 -R 200 -c 100 --pe 300 30 --bam --vcf
249	1000_200/7	-M 0.1 -R 200 -c 100 --pe 300 30 --bam --vcf
251	1000_200/8	-M 0.1 -R 200 -c 100 --pe 300 30 --bam --vcf
254	1000_200/9	-M 0.1 -R 200 -c 100 --pe 300 30 --bam --vcf
255	1000_200/10	-M 0.1 -R 200 -c 100 --pe 300 30 --bam --vcf
257	1000_250/1	-M 0.1 -R 250 -c 100 --pe 300 30 --bam --vcf
259	1000_250/2	-M 0.1 -R 250 -c 100 --pe 300 30 --bam --vcf
261	1000_250/3	-M 0.1 -R 250 -c 100 --pe 300 30 --bam --vcf
262	1000_250/4	-M 0.1 -R 250 -c 100 --pe 300 30 --bam --vcf
267	1000_250/5	-M 0.1 -R 250 -c 100 --pe 300 30 --bam --vcf
268	1000_250/6	-M 0.1 -R 250 -c 100 --pe 300 30 --bam --vcf
269	1000_250/7	-M 0.1 -R 250 -c 100 --pe 300 30 --bam --vcf
274	1000_250/8	-M 0.1 -R 250 -c 100 --pe 300 30 --bam --vcf
283	1000_250/9	-M 0.1 -R 250 -c 100 --pe 300 30 --bam --vcf
287	1000_250/10	-M 0.1 -R 250 -c 100 --pe 300 30 --bam --vcf
288	300_50/1	-M 0.1 -R 50 -c 30 --pe 300 30 --bam --vcf
291	300_50/2	-M 0.1 -R 50 -c 30 --pe 300 30 --bam --vcf
292	300_50/3	-M 0.1 -R 50 -c 30 --pe 300 30 --bam --vcf
293	300_50/4	-M 0.1 -R 50 -c 30 --pe 300 30 --bam --vcf
294	300_50/5	-M 0.1 -R 50 -c 30 --pe 300 30 --bam --vcf
295	300_50/6	-M 0.1 -R 50 -c 30 --pe 300 30 --bam --vcf
300	300_50/7	-M 0.1 -R 50 -c 30 --pe 300 30 --bam --vcf
305	300_50/8	-M 0.1 -R 50 -c 30 --pe 300 30 --bam --vcf
314	300_50/9	-M 0.1 -R 50 -c 30 --pe 300 30 --bam --vcf
315	300_50/10	-M 0.1 -R 50 -c 30 --pe 300 30 --bam --vcf
318	300_75/1	-M 0.1 -R 75 -c 30 --pe 300 30 --bam --vcf
320	300_75/2	-M 0.1 -R 75 -c 30 --pe 300 30 --bam --vcf
322	300_75/3	-M 0.1 -R 75 -c 30 --pe 300 30 --bam --vcf
325	300_75/4	-M 0.1 -R 75 -c 30 --pe 300 30 --bam --vcf

327	300_75/5	-M 0.1 -R 75 -c 30 --pe 300 30 --bam --vcf
329	300_75/6	-M 0.1 -R 75 -c 30 --pe 300 30 --bam --vcf
331	300_75/7	-M 0.1 -R 75 -c 30 --pe 300 30 --bam --vcf
332	300_75/8	-M 0.1 -R 75 -c 30 --pe 300 30 --bam --vcf
336	300_75/9	-M 0.1 -R 75 -c 30 --pe 300 30 --bam --vcf
339	300_75/10	-M 0.1 -R 75 -c 30 --pe 300 30 --bam --vcf
340	300_100/1	-M 0.1 -R 100 -c 30 --pe 300 30 --bam --vcf
341	300_100/2	-M 0.1 -R 100 -c 30 --pe 300 30 --bam --vcf
345	300_100/3	-M 0.1 -R 100 -c 30 --pe 300 30 --bam --vcf
347	300_100/4	-M 0.1 -R 100 -c 30 --pe 300 30 --bam --vcf
350	300_100/5	-M 0.1 -R 100 -c 30 --pe 300 30 --bam --vcf
352	300_100/6	-M 0.1 -R 100 -c 30 --pe 300 30 --bam --vcf
353	300_100/7	-M 0.1 -R 100 -c 30 --pe 300 30 --bam --vcf
356	300_100/8	-M 0.1 -R 100 -c 30 --pe 300 30 --bam --vcf
358	300_100/9	-M 0.1 -R 100 -c 30 --pe 300 30 --bam --vcf
359	300_100/10	-M 0.1 -R 100 -c 30 --pe 300 30 --bam --vcf
363	300_300/1	-M 0.1 -R 300 -c 30 --pe 300 30 --bam --vcf
364	300_300/2	-M 0.1 -R 300 -c 30 --pe 300 30 --bam --vcf
366	300_300/3	-M 0.1 -R 300 -c 30 --pe 300 30 --bam --vcf
369	300_300/4	-M 0.1 -R 300 -c 30 --pe 300 30 --bam --vcf
370	300_300/5	-M 0.1 -R 300 -c 30 --pe 300 30 --bam --vcf
371	300_300/6	-M 0.1 -R 300 -c 30 --pe 300 30 --bam --vcf
372	300_300/7	-M 0.1 -R 300 -c 30 --pe 300 30 --bam --vcf
375	300_300/8	-M 0.1 -R 300 -c 30 --pe 300 30 --bam --vcf
378	300_300/9	-M 0.1 -R 300 -c 30 --pe 300 30 --bam --vcf
380	300_300/10	-M 0.1 -R 300 -c 30 --pe 300 30 --bam --vcf
381	700_50/1	-M 0.1 -R 50 -c 70 --pe 300 30 --bam --vcf
386	700_50/2	-M 0.1 -R 50 -c 70 --pe 300 30 --bam --vcf
388	700_50/3	-M 0.1 -R 50 -c 70 --pe 300 30 --bam --vcf
389	700_50/4	-M 0.1 -R 50 -c 70 --pe 300 30 --bam --vcf
391	700_50/5	-M 0.1 -R 50 -c 70 --pe 300 30 --bam --vcf
395	700_50/6	-M 0.1 -R 50 -c 70 --pe 300 30 --bam --vcf

398	700_50/7	-M 0.1 -R 50 -c 70 --pe 300 30 --bam --vcf
399	700_50/8	-M 0.1 -R 50 -c 70 --pe 300 30 --bam --vcf
403	700_50/9	-M 0.1 -R 50 -c 70 --pe 300 30 --bam --vcf
405	700_50/10	-M 0.1 -R 50 -c 70 --pe 300 30 --bam --vcf
406	700_75/1	-M 0.1 -R 75 -c 70 --pe 300 30 --bam --vcf
410	700_75/2	-M 0.1 -R 75 -c 70 --pe 300 30 --bam --vcf
411	700_75/3	-M 0.1 -R 75 -c 70 --pe 300 30 --bam --vcf
418	700_75/4	-M 0.1 -R 75 -c 70 --pe 300 30 --bam --vcf
419	700_75/5	-M 0.1 -R 75 -c 70 --pe 300 30 --bam --vcf
421	700_75/6	-M 0.1 -R 75 -c 70 --pe 300 30 --bam --vcf
422	700_75/7	-M 0.1 -R 75 -c 70 --pe 300 30 --bam --vcf
425	700_75/8	-M 0.1 -R 75 -c 70 --pe 300 30 --bam --vcf
429	700_75/9	-M 0.1 -R 75 -c 70 --pe 300 30 --bam --vcf
431	700_75/10	-M 0.1 -R 75 -c 70 --pe 300 30 --bam --vcf
433	700_100/1	-M 0.1 -R 100 -c 70 --pe 300 30 --bam --vcf
435	700_100/2	-M 0.1 -R 100 -c 70 --pe 300 30 --bam --vcf
437	700_100/3	-M 0.1 -R 100 -c 70 --pe 300 30 --bam --vcf
438	700_100/4	-M 0.1 -R 100 -c 70 --pe 300 30 --bam --vcf
439	700_100/5	-M 0.1 -R 100 -c 70 --pe 300 30 --bam --vcf
440	700_100/6	-M 0.1 -R 100 -c 70 --pe 300 30 --bam --vcf
442	700_100/7	-M 0.1 -R 100 -c 70 --pe 300 30 --bam --vcf
447	700_100/8	-M 0.1 -R 100 -c 70 --pe 300 30 --bam --vcf
451	700_100/9	-M 0.1 -R 100 -c 70 --pe 300 30 --bam --vcf
453	700_100/10	-M 0.1 -R 100 -c 70 --pe 300 30 --bam --vcf
454	700_300/1	-M 0.1 -R 300 -c 70 --pe 300 30 --bam --vcf
455	700_300/2	-M 0.1 -R 300 -c 70 --pe 300 30 --bam --vcf
456	700_300/3	-M 0.1 -R 300 -c 70 --pe 300 30 --bam --vcf
457	700_300/4	-M 0.1 -R 300 -c 70 --pe 300 30 --bam --vcf
459	700_300/5	-M 0.1 -R 300 -c 70 --pe 300 30 --bam --vcf
461	700_300/6	-M 0.1 -R 300 -c 70 --pe 300 30 --bam --vcf
462	700_300/7	-M 0.1 -R 300 -c 70 --pe 300 30 --bam --vcf
463	700_300/8	-M 0.1 -R 300 -c 70 --pe 300 30 --bam --vcf

464	700_300/9	-M 0.1 -R 300 -c 70 --pe 300 30 --bam --vcf
465	700_300/10	-M 0.1 -R 300 -c 70 --pe 300 30 --bam --vcf
466	3000_50/1	-M 0.1 -R 50 -c 300 --pe 300 30 --bam --vcf
470	3000_50/2	-M 0.1 -R 50 -c 300 --pe 300 30 --bam --vcf
473	3000_50/3	-M 0.1 -R 50 -c 300 --pe 300 30 --bam --vcf
475	3000_50/4	-M 0.1 -R 50 -c 300 --pe 300 30 --bam --vcf
477	3000_50/5	-M 0.1 -R 50 -c 300 --pe 300 30 --bam --vcf
478	3000_50/6	-M 0.1 -R 50 -c 300 --pe 300 30 --bam --vcf
480	3000_50/7	-M 0.1 -R 50 -c 300 --pe 300 30 --bam --vcf
481	3000_50/8	-M 0.1 -R 50 -c 300 --pe 300 30 --bam --vcf
482	3000_50/9	-M 0.1 -R 50 -c 300 --pe 300 30 --bam --vcf
483	3000_50/10	-M 0.1 -R 50 -c 300 --pe 300 30 --bam --vcf
484	3000_75/1	-M 0.1 -R 75 -c 300 --pe 300 30 --bam --vcf
489	3000_75/2	-M 0.1 -R 75 -c 300 --pe 300 30 --bam --vcf
492	3000_75/3	-M 0.1 -R 75 -c 300 --pe 300 30 --bam --vcf
494	3000_75/4	-M 0.1 -R 75 -c 300 --pe 300 30 --bam --vcf
496	3000_75/5	-M 0.1 -R 75 -c 300 --pe 300 30 --bam --vcf
500	3000_75/6	-M 0.1 -R 75 -c 300 --pe 300 30 --bam --vcf
501	3000_75/7	-M 0.1 -R 75 -c 300 --pe 300 30 --bam --vcf
503	3000_75/8	-M 0.1 -R 75 -c 300 --pe 300 30 --bam --vcf
504	3000_75/9	-M 0.1 -R 75 -c 300 --pe 300 30 --bam --vcf
505	3000_75/10	-M 0.1 -R 75 -c 300 --pe 300 30 --bam --vcf
507	3000_100/1	-M 0.1 -R 100 -c 300 --pe 300 30 --bam --vcf
513	3000_100/2	-M 0.1 -R 100 -c 300 --pe 300 30 --bam --vcf
514	3000_100/3	-M 0.1 -R 100 -c 300 --pe 300 30 --bam --vcf
515	3000_100/4	-M 0.1 -R 100 -c 300 --pe 300 30 --bam --vcf
516	3000_100/5	-M 0.1 -R 100 -c 300 --pe 300 30 --bam --vcf
517	3000_100/6	-M 0.1 -R 100 -c 300 --pe 300 30 --bam --vcf
519	3000_100/7	-M 0.1 -R 100 -c 300 --pe 300 30 --bam --vcf
521	3000_100/8	-M 0.1 -R 100 -c 300 --pe 300 30 --bam --vcf
524	3000_100/9	-M 0.1 -R 100 -c 300 --pe 300 30 --bam --vcf
525	3000_100/10	-M 0.1 -R 100 -c 300 --pe 300 30 --bam --vcf

526	3000_300/1	-M 0.1 -R 300 -c 300 --pe 300 30 --bam --vcf
528	3000_300/2	-M 0.1 -R 300 -c 300 --pe 300 30 --bam --vcf
530	3000_300/3	-M 0.1 -R 300 -c 300 --pe 300 30 --bam --vcf
531	3000_300/4	-M 0.1 -R 300 -c 300 --pe 300 30 --bam --vcf
533	3000_300/5	-M 0.1 -R 300 -c 300 --pe 300 30 --bam --vcf
536	3000_300/6	-M 0.1 -R 300 -c 300 --pe 300 30 --bam --vcf
537	3000_300/7	-M 0.1 -R 300 -c 300 --pe 300 30 --bam --vcf
541	3000_300/8	-M 0.1 -R 300 -c 300 --pe 300 30 --bam --vcf
542	3000_300/9	-M 0.1 -R 300 -c 300 --pe 300 30 --bam --vcf
544	3000_300/10	-M 0.1 -R 300 -c 300 --pe 300 30 --bam --vcf
546	5000_50/1	-M 0.1 -R 50 -c 500 --pe 300 30 --bam --vcf
549	5000_50/2	-M 0.1 -R 50 -c 500 --pe 300 30 --bam --vcf
550	5000_50/3	-M 0.1 -R 50 -c 500 --pe 300 30 --bam --vcf
552	5000_50/4	-M 0.1 -R 50 -c 500 --pe 300 30 --bam --vcf
553	5000_50/5	-M 0.1 -R 50 -c 500 --pe 300 30 --bam --vcf
556	5000_50/6	-M 0.1 -R 50 -c 500 --pe 300 30 --bam --vcf
558	5000_50/7	-M 0.1 -R 50 -c 500 --pe 300 30 --bam --vcf
560	5000_50/8	-M 0.1 -R 50 -c 500 --pe 300 30 --bam --vcf
561	5000_50/9	-M 0.1 -R 50 -c 500 --pe 300 30 --bam --vcf
562	5000_50/10	-M 0.1 -R 50 -c 500 --pe 300 30 --bam --vcf
564	5000_75/1	-M 0.1 -R 75 -c 500 --pe 300 30 --bam --vcf
567	5000_75/2	-M 0.1 -R 75 -c 500 --pe 300 30 --bam --vcf
570	5000_75/3	-M 0.1 -R 75 -c 500 --pe 300 30 --bam --vcf
571	5000_75/4	-M 0.1 -R 75 -c 500 --pe 300 30 --bam --vcf
572	5000_75/5	-M 0.1 -R 75 -c 500 --pe 300 30 --bam --vcf
573	5000_75/6	-M 0.1 -R 75 -c 500 --pe 300 30 --bam --vcf
574	5000_75/7	-M 0.1 -R 75 -c 500 --pe 300 30 --bam --vcf
576	5000_75/8	-M 0.1 -R 75 -c 500 --pe 300 30 --bam --vcf
578	5000_75/9	-M 0.1 -R 75 -c 500 --pe 300 30 --bam --vcf
579	5000_75/10	-M 0.1 -R 75 -c 500 --pe 300 30 --bam --vcf
580	5000_100/1	-M 0.1 -R 100 -c 500 --pe 300 30 --bam --vcf
581	5000_100/2	-M 0.1 -R 100 -c 500 --pe 300 30 --bam --vcf

585	5000_100/3	-M 0.1 -R 100 -c 500 --pe 300 30 --bam --vcf
587	5000_100/4	-M 0.1 -R 100 -c 500 --pe 300 30 --bam --vcf
589	5000_100/5	-M 0.1 -R 100 -c 500 --pe 300 30 --bam --vcf
591	5000_100/6	-M 0.1 -R 100 -c 500 --pe 300 30 --bam --vcf
592	5000_100/7	-M 0.1 -R 100 -c 500 --pe 300 30 --bam --vcf
593	5000_100/8	-M 0.1 -R 100 -c 500 --pe 300 30 --bam --vcf
596	5000_100/9	-M 0.1 -R 100 -c 500 --pe 300 30 --bam --vcf
598	5000_100/10	-M 0.1 -R 100 -c 500 --pe 300 30 --bam --vcf
600	5000_300/1	-M 0.1 -R 300 -c 500 --pe 300 30 --bam --vcf
603	5000_300/2	-M 0.1 -R 300 -c 500 --pe 300 30 --bam --vcf
604	5000_300/3	-M 0.1 -R 300 -c 500 --pe 300 30 --bam --vcf
605	5000_300/4	-M 0.1 -R 300 -c 500 --pe 300 30 --bam --vcf
607	5000_300/5	-M 0.1 -R 300 -c 500 --pe 300 30 --bam --vcf
611	5000_300/6	-M 0.1 -R 300 -c 500 --pe 300 30 --bam --vcf
615	5000_300/7	-M 0.1 -R 300 -c 500 --pe 300 30 --bam --vcf
616	5000_300/8	-M 0.1 -R 300 -c 500 --pe 300 30 --bam --vcf
619	5000_300/9	-M 0.1 -R 300 -c 500 --pe 300 30 --bam --vcf
620	5000_300/10	-M 0.1 -R 300 -c 500 --pe 300 30 --bam --vcf

S3 Comparison of run times

We calculated the total runtime for the generation and variant calling process for one of the most computationally heavy datasets (i.e. that of 5000x coverage, which contained a total of 620,642 reads). The dataset generation step required approximately 6 minutes. For the variant calling stage, the elapsed times were as follows: Mutect2 - 50 minutes, VarDict - 12 minutes, LoFreq - 12 minutes VarScan2 - 3 minutes and FreeBayes - 2 minutes.
Furthermore, we recorded the time needed for our R scripts to execute the benchmarking as well as the visualization for 10 datasets. As expected, processing time increased with the number of reads generated at higher coverage levels with a mean *S4BR.R* run time of 25.22sec. On average, the visualization script (*S4BR_plot.R*) required more time to be executed (equal to 39.1 sec) and was not affected by dataset depth. Values for the run times comparison are presented in tables of this section below.
All experiments were performed on a desktop system equipped with an Intel(R) Xeon(R) CPU E5-2673 v3 @ 2.40GHz and 32 GB of RAM, running in WSL2 within a conda environment.
In the following tables the time duration that was required for each R script to perform the benchmark are presented. In each cell there are 2 values, the first for the analysis from *S4BR.R* script and the second for the visualization script *S4BR_plot.R*. All values of the tables are in seconds.

Values for datasets of fixed read length at 150bp and varying coverage.

	**Time for Coverage Datasets [sec]**				
**Caller**	**300x**	**700x**	**1000x**	**3000x**	**5000x**
Mutect2	16, 36	32, 37	31, 38	32, 40	17, 44
FreeBayes	25, 32	29, 35	32, 35	45, 38	56, 40
VarDict	23, 37	23, 40	24, 37	25, 43	25, 43
VarScan2	21, 38	21, 42	22, 38	23, 41	23, 43
LoFreq	19, 35	18, 37	18, 38	18, 42	19, 42

Values for datasets of fixed coverage at 1000x and varying read length.

	**Time for Read Length Datasets [sec]**				
**Caller**	**50**	**75**	**100**	**150**	**300**
Mutect2	19, 42	35, 43	33, 39	31, 38	31, 42
FreeBayes	34, 38	34, 39	14, 35	32, 35	33, 36
VarDict	26, 41	25, 40	24, 39	24, 37	24, 40
VarScan2	23, 42	22, 41	23, 41	22, 38	21, 40
LoFreq	18, 40	20, 40	19, 40	18, 38	19, 40

S4 Recall and precision heat maps data

Coverage	Read Length	Caller	True Variants Recall	True Variants Precision	Noise Recall	Noise Precision	Indel Recall	Indel Precision
300	50	FreeBayes	0.4790286976	1	0.1964853751	1	0	0
300	50	Mutect2	0.5960264901	1	0.2739545349	0.9987271956	0.1575673808	0.3937823834
300	50	VarDict	0.7902869757	1	0.4120179998	1	0.3144436766	0.4945652174
300	50	VarScan2	0.9624724062	1	0.4833191093	0.9980774	0.1734623359	0.6972222222
300	50	LoFreq	0.8454746137	1	0.4797889673	1	0.3324118867	0.7189835575
300	75	FreeBayes	0.5382585752	1	0.1899984506	1	0	0
300	75	Mutect2	0.654353562	1	0.2737062287	0.9994342291	0.1739441661	0.4516728625
300	75	VarDict	0.7862796834	1	0.4199333747	1	0.340730136	0.5010526316
300	75	VarScan2	0.944591029	1	0.488805392	0.9977071	0.1875447387	0.7061994609
300	75	LoFreq	0.8179419525	1	0.4950805702	1	0.3772369363	0.7289073306
300	100	FreeBayes	0.4814814815	1	0.1784200385	1	0	0
300	100	Mutect2	0.5925925926	1	0.2489017341	0.9993810924	0.1681136543	0.4243027888
300	100	VarDict	0.7916666667	1	0.4203853565	1	0.3464877664	0.4960451977
300	100	VarScan2	0.9722222222	1	0.4869749518	0.9981044	0.1933701657	0.6940509915
300	100	LoFreq	0.8333333333	1	0.4972254335	1	0.3883188635	0.7172011662
300	150	FreeBayes	0.179028133	1	0.2350206815	1	NaN	0
300	150	Mutect2	0.5421994885	1	0.2033090408	0.9986455108	NaN	0
300	150	VarDict	0.7493606138	1	0.4263935395	1	NaN	0

300	150	VarScan2	0.9616368286	1	0.4962379358	0.9969136	NaN	0
300	150	LoFreq	0.7979539642	1	0.5119952728	1	NaN	0
300	300	FreeBayes	0.4073226545	1	0.1632183908	1	0	0
300	300	Mutect2	0.4027459954	1	0.1648158971	0.9985835694	0.0856685348 3	0.41960784 31
300	300	VarDict	0.7551487414	1	0.413559322	1	0.3634907926	0.49509269 36
300	300	VarScan2	0.9336384439	1	0.4553672316	0.9961643	0.2025620496	0.69315068 49
300	300	LoFreq	0.76201373	1	0.4812000779	1	0.4163330665	0.72727272 73
700	50	FreeBayes	0.564738292	1	0.1529675314	1	0	0
700	50	Mutect2	0.650137741	1	0.2080037613	0.9996012759	0.1239749156	0.44695652 17
700	50	VarDict	0.7851239669	1	0.307622103	1	0.2373371925	0.51196670 14
700	50	VarScan2	0.9696969697	1	0.3904806682	0.9988681	0.1394114809	0.71712158 81
700	50	LoFreq	0.782369146	1	0.3767907517	1	0.3019778099	0.72706155 63
700	75	FreeBayes	0.5135135135	1	0.1441946023	1	0	0
700	75	Mutect2	0.6636636637	1	0.2095379293	0.9993452927	0.1248081841	0.40666666 67
700	75	VarDict	0.8108108108	1	0.3063998023	1	0.2347826087	0.49143468 95
700	75	VarScan2	0.96996997	1	0.3879965956	0.9983046	0.1324808184	0.7
700	75	LoFreq	0.8678678679	1	0.3703703704	1	0.3007672634	0.70250896 06
700	100	FreeBayes	0.4985590778	1	0.1406899994	1	0	0
700	100	Mutect2	0.6224783862	1	0.1833435021	0.9992442563	0.1090204759	0.38551859 1
700	100	VarDict	0.8040345821	1	0.3068944478	1	0.2318760376	0.46196251 38
700	100	VarScan2	0.976945245	1	0.3906206667	0.9982990	0.1433314887	0.71546961 33

700	100	LoFreq	0.8069164265	1	0.3775583782	1	0.3220807969	0.7256857855
700	150	FreeBayes	0.2	1	0.232408735	1	0	0
700	150	Mutect2	0.6356164384	1	0.1622918275	0.9989816701	0.1155356141	0.5036674817
700	150	VarDict	0.8082191781	1	0.3059170619	1	0.2697700505	0.5020876827
700	150	VarScan2	0.9808219178	1	0.3874765634	0.9980115	0.1463825014	0.7073170732
700	150	LoFreq	0.8575342466	1	0.3761166869	1	0.3499719574	0.7281213536
700	300	FreeBayes	0.4444444444	1	0.1241542349	1	0	0
700	300	Mutect2	0.5126262626	1	0.1280671721	0.9991520034	0.08384918402	0.4570552147
700	300	VarDict	0.7929292929	1	0.2974375696	1	0.2718064153	0.488372093
700	300	VarScan2	0.946969697	1	0.334990897	0.9961215	0.1699493528	0.7365853659
700	300	LoFreq	0.7626262626	1	0.3642835793	1	0.3680360158	0.7323628219
1000	50	FreeBayes	0.1338028169	1	0.2445815955	1	0	0
1000	50	Mutect2	0.6408450704	1	0.1852744762	0.9998698933	0.09883268482	0.4247491639
1000	50	VarDict	0.7922535211	1	0.2741387208	1	0.1789883268	0.487804878
1000	50	VarScan2	0.9647887324	1	0.3588080716	0.9985910	0.1112840467	0.7222222222
1000	50	LoFreq	0.7992957746	1	0.3311314159	1	0.246692607	0.7363530778
1000	75	FreeBayes	0.1214057508	1	0.2372572087	1	0	0
1000	75	Mutect2	0.6453674121	1	0.1705121126	0.9992964683	0.1012826183	0.4126126126
1000	75	VarDict	0.8274760383	1	0.2709898922	1	0.1941618753	0.4740820734
1000	75	VarScan2	0.9680511182	1	0.3599433387	0.9982023	0.125608138	0.7415143603

1000	75	LoFreq	0.8178913738	1	0.3301481357	1	0.2649270234	0.7139451728
1000	100	FreeBayes	0.0369318181 8	1	0.0005067567 568	1	0	Nan
1000	100	Mutect2	0.5994318182	1	0.1659749035	0.9995640169	0.1077283372	0.4466019417
1000	100	VarDict	0.8039772727	1	0.2746380309	1	0.2149882904	0.4751552795
1000	100	VarScan2	0.9659090909	1	0.3630067568	0.9988049	0.1269320843	0.7226666667
1000	100	LoFreq	0.84375	1	0.3306949807	1	0.2861826698	0.7112922002
1000	150	FreeBayes	0.1743421053	1	0.2405263916	1	0	0
1000	150	Mutect2	0.6085526316	1	0.1454541088	0.9993400429	0.0845892075 8	0.4055944056
1000	150	VarDict	0.8453947368	1	0.2711205033	1	0.2124453087	0.4580712788
1000	150	VarScan2	0.9769736842	1	0.3586763364	0.9983290	0.1317452601	0.7150395778
1000	150	LoFreq	0.8453947368	1	0.3287306085	1	0.2897423432	0.7163461538
1000	300	FreeBayes	0.1485714286	1	0.236836983	1	0	0
1000	300	Mutect2	0.4342857143	1	0.1148418491	0.9991532599	0.0695792880 3	0.446366782
1000	300	VarDict	0.7542857143	1	0.272676399	1	0.240021575	0.4674369748
1000	300	VarScan2	0.9428571429	1	0.3133090024	0.9965947	0.1574973031	0.7411167513
1000	300	LoFreq	0.7628571429	1	0.332676399	1	0.3203883495	0.7071428571
3000	50	FreeBayes	0.6271186441	1	0.1326920225	1	0	0
3000	50	Mutect2	0.6271186441	1	0.1419780546	0.9998694687	0.0540958268 9	0.4166666667
3000	50	VarDict	0.8305084746	1	0.2173413405	1	0.1022300729	0.4822916667
3000	50	VarScan2	0.9717514124	1	0.324992586	0.9994300	0.0713181717 8	0.7307692308

3000	50	LoFreq	0.7853107345	1	0.2557273132	1	0.1488187238	0.7255113025
3000	75	FreeBayes	0.6216216216	1	0.1247753007	1	0	0
3000	75	Mutect2	0.6324324324	1	0.1302792758	1	0.0563124432 3	0.4335664336
3000	75	VarDict	0.827027027	1	0.2182687497	1	0.1101271571	0.4830677291
3000	75	VarScan2	0.9621621622	1	0.3274772521	0.9993779	0.0676657584	0.7095238095
3000	75	LoFreq	0.8162162162	1	0.2597431478	1	0.1537238874	0.7148891235
3000	100	FreeBayes	0.4795918367	1	0.1200636589	1	0	0
3000	100	Mutect2	0.637755102	1	0.1308523632	0.9998585973	0.0489080982 7	0.4150579151
3000	100	VarDict	0.8367346939	1	0.2161441948	1	0.1071428571	0.4667988107
3000	100	VarScan2	0.943877551	1	0.327787853	0.9996050	0.0671064604 2	0.7195121951
3000	100	LoFreq	0.7959183673	1	0.2601502646	1	0.1505914468	0.725877193
3000	150	FreeBayes	0.0256410256 4	1	0.1477460424	1	0	0
3000	150	Mutect2	0.5641025641	1	0.1132082475	1	0.0385099421 1	0.3713592233
3000	150	VarDict	0.8205128205	1	0.2191229145	1	0.1150264284	0.4579158317
3000	150	VarScan2	0.9615384615	1	0.3281554479	0.9995478	0.0739994966	0.7188264059
3000	150	LoFreq	0.7820512821	1	0.2609728486	1	0.1620941354	0.7351598174
3000	300	FreeBayes	0.4059405941	1	0.0964809547 7	1	0	0
3000	300	Mutect2	0.5495049505	1	0.0933021025	0.9998007968	0.0382905138 3	0.4492753623
3000	300	VarDict	0.7871287129	1	0.2115888684	1	0.1299407115	0.4816849817

3000	300	VarScan2	0.9405940594	1	0.2785492536	0.9968068	0.09412055336	0.7589641434
3000	300	LoFreq	0.7772277228	1	0.2583979328	1	0.1776185771	0.7536687631
5000	50	FreeBayes	0.6202531646	1	0.1415924484	1	0	0
5000	50	Mutect2	0.7215189873	1	0.132970686	1	0.04030183502	0.4065743945
5000	50	VarDict	0.8101265823	1	0.2085222121	1	0.07614474361	0.458677686
5000	50	VarScan2	0.9240506329	1	0.3257426271	0.9997272	0.0519636426	0.7163120567
5000	50	LoFreq	0.7721518987	1	0.2514888095	1	0.1166180758	0.7188160677
5000	75	FreeBayes	0.5934065934	1	0.1342783275	1	0	0
5000	75	Mutect2	0.5384615385	1	0.1317286262	1	0.0382209868	0.3886925795
5000	75	VarDict	0.8351648352	1	0.2086119283	1	0.07956914524	0.4815983176
5000	75	VarScan2	0.9450549451	1	0.3262726219	0.9995630	0.05055594163	0.6978417266
5000	75	LoFreq	0.7802197802	1	0.2517428903	1	0.1162265462	0.730349345
5000	100	FreeBayes	0.6341463415	1	0.1269776226	1	0	0
5000	100	Mutect2	0.6829268293	1	0.128313854	0.999861169	0.03926568078	0.4317757009
5000	100	VarDict	0.8536585366	1	0.2080779647	1	0.08431072582	0.4741873805
5000	100	VarScan2	0.987804878	1	0.3269847491	0.9996732	0.04776474588	0.7025
5000	100	LoFreq	0.8170731707	1	0.2517994584	1	0.1154173041	0.7316810345
5000	150	FreeBayes	0.03370786517	1	0.04878962485	1	0	0
5000	150	Mutect2	0.5617977528	1	0.1075155645	1	0.03143287076	0.432183908
5000	150	VarDict	0.8651685393	1	0.2078420179	1	0.08527002174	0.4636363636

5000	150	VarScan2	0.9550561798	1	0.3260074567	0.9996718	0.0509948169<br>2	0.74029126<br>21
5000	150	LoFreq	0.8314606742	1	0.2522967694	1	0.1203812072	0.75630252<br>1
5000	300	FreeBayes	0.4774774775	1	0.1005273979	1	0	0
5000	300	Mutect2	0.4864864865	1	0.0964691159<br>4	1	0.0273116744	0.42737430<br>17
5000	300	VarDict	0.8288288288	1	0.2063287745	1	0.090860407	0.47042513<br>86
5000	300	VarScan2	0.954954955	1	0.2936801645	0.9959379	0.0740806854<br>7	0.78449905<br>48
5000	300	LoFreq	0.7927927928	1	0.2522034504	1	0.1212067119	0.7578125

S5 AF bin plot data

Group	Caller	PPV	Recall	Type
AF ≤ 0.001	Mutect2	NaN	0	Indel
AF ≤ 0.001	FreeBayes	0	0	Indel
AF ≤ 0.001	VarDict	0.1882845188	0.002179071231	Indel
AF ≤ 0.001	VarScan2	0.9117959617	0.02062797519	Indel
AF ≤ 0.001	LoFreq	NaN	0	Indel
0.001 < AF ≤ 0.002	Mutect2	NaN	0	Indel
0.001 < AF ≤ 0.002	FreeBayes	NaN	0	Indel
0.001 < AF ≤ 0.002	VarDict	0.2046332046	0.005536982867	Indel
0.001 < AF ≤ 0.002	VarScan2	0.7741935484	0.0252286345	Indel
0.001 < AF ≤ 0.002	LoFreq	1	0.007645580226	Indel
0.002 < AF ≤ 0.005	Mutect2	0.5882352941	0.003850596843	Indel
0.002 < AF ≤ 0.005	FreeBayes	NaN	0	Indel
0.002 < AF ≤ 0.005	VarDict	0.2386363636	0.03148425787	Indel
0.002 < AF ≤ 0.005	VarScan2	0.6804733728	0.0853432282	Indel
0.002 < AF ≤ 0.005	LoFreq	1	0.006172839506	Indel
0.005 < AF ≤ 0.01	Mutect2	0.3529411765	0.1951219512	Indel
0.005 < AF ≤ 0.01	FreeBayes	NaN	0	Indel
0.005 < AF ≤ 0.01	VarDict	0.3387533875	0.5580357143	Indel
0.005 < AF ≤ 0.01	VarScan2	0.5833333333	0.3157894737	Indel
0.005 < AF ≤ 0.01	LoFreq	1	0.0303030303	Indel
0.01 < AF ≤ 0.02	Mutect2	0.3886639676	0.6530612245	Indel
0.01 < AF ≤ 0.02	FreeBayes	NaN	0	Indel
0.01 < AF ≤ 0.02	VarDict	0.4248826291	0.8578199052	Indel
0.01 < AF ≤ 0.02	VarScan2	0.3409090909	0.2419354839	Indel
0.01 < AF ≤ 0.02	LoFreq	0.6666666667	0.4444444444	Indel
0.02 < AF ≤ 0.05	Mutect2	0.383910387	0.06781795287	Indel
0.02 < AF ≤ 0.05	FreeBayes	NaN	0	Indel
0.02 < AF ≤ 0.05	VarDict	0.4573323508	0.488738384	Indel
0.02 < AF ≤ 0.05	VarScan2	0.6684144295	0.2418057663	Indel
0.02 < AF ≤ 0.05	LoFreq	0.7262180974	0.6670613308	Indel

0.05 < AF ≤ 0.1	Mutect2	0.4227591904	0.2247228016	Indel
0.05 < AF ≤ 0.1	FreeBayes	NaN	0	Indel
0.05 < AF ≤ 0.1	VarDict	0.5057273769	0.4771941202	Indel
0.05 < AF ≤ 0.1	VarScan2	0.6787148594	0.2699988588	Indel
0.05 < AF ≤ 0.1	LoFreq	0.6713630057	0.6560967977	Indel
0.1 < AF ≤ 0.5	Mutect2	0.4077134986	0.9687814703	Indel
0.1 < AF ≤ 0.5	FreeBayes	0	0	Indel
0.1 < AF ≤ 0.5	VarDict	0.6923076923	0.5833333333	Indel
0.1 < AF ≤ 0.5	VarScan2	0.6875	0.3473684211	Indel
0.1 < AF ≤ 0.5	LoFreq	0.6727272727	0.6788990826	Indel
0.5 < AF ≤ 1	Mutect2	0.4137931034	1	Indel
0.5 < AF ≤ 1	FreeBayes	0	NaN	Indel
0.5 < AF ≤ 1	VarDict	NaN	NaN	Indel
0.5 < AF ≤ 1	VarScan2	NaN	NaN	Indel
0.5 < AF ≤ 1	LoFreq	NaN	NaN	Indel
AF ≤ 0.001	Mutect2	NaN	NaN	TV
AF ≤ 0.001	FreeBayes	NaN	NaN	TV
AF ≤ 0.001	VarDict	NaN	NaN	TV
AF ≤ 0.001	VarScan2	NaN	NaN	TV
AF ≤ 0.001	LoFreq	NaN	NaN	TV
0.001 < AF ≤ 0.002	Mutect2	NaN	NaN	TV
0.001 < AF ≤ 0.002	FreeBayes	NaN	NaN	TV
0.001 < AF ≤ 0.002	VarDict	NaN	NaN	TV
0.001 < AF ≤ 0.002	VarScan2	NaN	NaN	TV
0.001 < AF ≤ 0.002	LoFreq	NaN	NaN	TV
0.002 < AF ≤ 0.005	Mutect2	NaN	NaN	TV
0.002 < AF ≤ 0.005	FreeBayes	NaN	NaN	TV
0.002 < AF ≤ 0.005	VarDict	NaN	NaN	TV
0.002 < AF ≤ 0.005	VarScan2	NaN	NaN	TV
0.002 < AF ≤ 0.005	LoFreq	NaN	NaN	TV
0.005 < AF ≤ 0.01	Mutect2	NaN	NaN	TV
0.005 < AF ≤ 0.01	FreeBayes	NaN	NaN	TV

0.005 < AF ≤ 0.01	VarDict	NaN	NaN	TV
0.005 < AF ≤ 0.01	VarScan2	NaN	NaN	TV
0.005 < AF ≤ 0.01	LoFreq	NaN	NaN	TV
0.01 < AF ≤ 0.02	Mutect2	NaN	0	TV
0.01 < AF ≤ 0.02	FreeBayes	NaN	0	TV
0.01 < AF ≤ 0.02	VarDict	NaN	0	TV
0.01 < AF ≤ 0.02	VarScan2	NaN	0	TV
0.01 < AF ≤ 0.02	LoFreq	NaN	0	TV
0.02 < AF ≤ 0.05	Mutect2	1	0.2713178295	TV
0.02 < AF ≤ 0.05	FreeBayes	1	0.06976744186	TV
0.02 < AF ≤ 0.05	VarDict	1	0.5271317829	TV
0.02 < AF ≤ 0.05	VarScan2	1	0.7519379845	TV
0.02 < AF ≤ 0.05	LoFreq	1	0.6279069767	TV
0.05 < AF ≤ 0.1	Mutect2	1	0.4855244423	TV
0.05 < AF ≤ 0.1	FreeBayes	1	0.3939250119	TV
0.05 < AF ≤ 0.1	VarDict	1	0.7104888467	TV
0.05 < AF ≤ 0.1	VarScan2	1	0.9335548173	TV
0.05 < AF ≤ 0.1	LoFreq	1	0.7826293308	TV
0.1 < AF ≤ 0.5	Mutect2	1	0.6398790235	TV
0.1 < AF ≤ 0.5	FreeBayes	1	0.3555843595	TV
0.1 < AF ≤ 0.5	VarDict	1	0.8461870814	TV
0.1 < AF ≤ 0.5	VarScan2	1	0.9788291208	TV
0.1 < AF ≤ 0.5	LoFreq	1	0.8265284079	TV
0.5 < AF ≤ 1	Mutect2	NaN	0	TV
0.5 < AF ≤ 1	FreeBayes	1	1	TV
0.5 < AF ≤ 1	VarDict	NaN	0	TV
0.5 < AF ≤ 1	VarScan2	NaN	0	TV
0.5 < AF ≤ 1	LoFreq	NaN	0	TV
AF ≤ 0.001	Mutect2	NaN	0	Noise
AF ≤ 0.001	FreeBayes	1	0.4139151862	Noise
AF ≤ 0.001	VarDict	1	0.02178287409	Noise
AF ≤ 0.001	VarScan2	0.480373904	0.2808602799	Noise

AF ≤ 0.001	LoFreq	NaN	0	Noise
0.001 < AF ≤ 0.002	Mutect2	NaN	0	Noise
0.001 < AF ≤ 0.002	FreeBayes	NaN	0	Noise
0.001 < AF ≤ 0.002	VarDict	1	0.00552872907	Noise
0.001 < AF ≤ 0.002	VarScan2	0.9943172034	0.04144891748	Noise
0.001 < AF ≤ 0.002	LoFreq	NaN	0	Noise
0.002 < AF ≤ 0.005	Mutect2	1	0.0006244180314	Noise
0.002 < AF ≤ 0.005	FreeBayes	NaN	0	Noise
0.002 < AF ≤ 0.005	VarDict	1	0.02468248004	Noise
0.002 < AF ≤ 0.005	VarScan2	0.9934936679	0.05420430414	Noise
0.002 < AF ≤ 0.005	LoFreq	NaN	0	Noise
0.005 < AF ≤ 0.01	Mutect2	1	0.04449417495	Noise
0.005 < AF ≤ 0.01	FreeBayes	NaN	0	Noise
0.005 < AF ≤ 0.01	VarDict	1	0.1952786072	Noise
0.005 < AF ≤ 0.01	VarScan2	0.9789029536	0.07348166917	Noise
0.005 < AF ≤ 0.01	LoFreq	NaN	0	Noise
0.01 < AF ≤ 0.02	Mutect2	0.9995791246	0.6165628245	Noise
0.01 < AF ≤ 0.02	FreeBayes	NaN	0	Noise
0.01 < AF ≤ 0.02	VarDict	1	0.4628942486	Noise
0.01 < AF ≤ 0.02	VarScan2	0.9960988296	0.4234383637	Noise
0.01 < AF ≤ 0.02	LoFreq	1	0.007105943152	Noise
0.02 < AF ≤ 0.05	Mutect2	0.999660427	0.2299116513	Noise
0.02 < AF ≤ 0.05	FreeBayes	NaN	0	Noise
0.02 < AF ≤ 0.05	VarDict	1	0.7797355284	Noise
0.02 < AF ≤ 0.05	VarScan2	0.9988862444	0.7643882477	Noise
0.02 < AF ≤ 0.05	LoFreq	1	0.9374253849	Noise
0.05 < AF ≤ 0.1	Mutect2	0.9995701911	0.3854701236	Noise
0.05 < AF ≤ 0.1	FreeBayes	NaN	0	Noise
0.05 < AF ≤ 0.1	VarDict	1	0.7589168679	Noise
0.05 < AF ≤ 0.1	VarScan2	0.9989328877	0.810815529	Noise
0.05 < AF ≤ 0.1	LoFreq	1	0.9801715941	Noise
0.1 < AF ≤ 0.5	Mutect2	0.9994738955	0.8220430659	Noise

0.1 < AF ≤ 0.5	FreeBayes	1	0.3485257275	Noise
0.1 < AF ≤ 0.5	VarDict	1	0.8385672228	Noise
0.1 < AF ≤ 0.5	VarScan2	0.9999021271	0.9834114099	Noise
0.1 < AF ≤ 0.5	LoFreq	1	0.9982305246	Noise
0.5 < AF ≤ 1	Mutect2	0.9994394619	1	Noise
0.5 < AF ≤ 1	FreeBayes	NaN	NaN	Noise
0.5 < AF ≤ 1	VarDict	NaN	NaN	Noise
0.5 < AF ≤ 1	VarScan2	NaN	NaN	Noise
0.5 < AF ≤ 1	LoFreq	NaN	NaN	Noise

S6 ΔAF heatmap data

Coverage	Read Length	Caller	mean	std
300	100	FreeBayes	0.000204	0.001023
300	100	LoFreq	-0.00043	0.001639
1000	100	LoFreq	-0.00064	0.001739
300	100	Mutect2	0.036572	0.101344
300	100	VarDict	-0.02062	0.025935
1000	100	VarDict	-0.02311	0.028972
300	100	VarScan2	-0.00661	0.009113
1000	100	VarScan2	-0.00647	0.007832
3000	100	LoFreq	-0.00066	0.001772
3000	100	Mutect2	0.026652	0.095789
3000	100	VarDict	-0.02602	0.027735
3000	100	VarScan2	-0.00624	0.006677
700	150	FreeBayes	-3.69E-05	0.001379
700	150	LoFreq	-0.00049	0.00157
700	150	Mutect2	0.051832	0.113807
700	150	VarDict	-0.02368	0.02915
700	150	VarScan2	-0.00485	0.008979
300	150	LoFreq	-0.0006	0.00209
300	150	Mutect2	0.079683	0.138371
300	150	VarDict	-0.02266	0.028925
300	150	VarScan2	-0.00369	0.009473
700	300	LoFreq	-0.00046	0.001526
700	300	VarDict	-0.02225	0.029701
700	300	VarScan2	-0.00177	0.006548
300	300	FreeBayes	-0.00015	0.002738
300	300	LoFreq	-0.00034	0.001367
700	50	LoFreq	-0.00056	0.001736
300	300	Mutect2	0.121956	0.165195
700	50	Mutect2	0.016178	0.079268

300	300	VarDict	-0.02093	0.02733
300	300	VarScan2	-0.00281	0.010404
700	50	VarScan2	-0.01181	0.008014
300	50	FreeBayes	5.21E-05	0.001003
300	50	LoFreq	-0.00048	0.001566
1000	150	LoFreq	-0.00041	0.00132
300	50	Mutect2	0.013618	0.081686
1000	150	Mutect2	0.070566	0.113121
300	50	VarDict	-0.02444	0.029853
1000	150	VarDict	-0.02023	0.026633
300	50	VarScan2	-0.0128	0.013049
1000	150	VarScan2	-0.00366	0.006197
700	50	FreeBayes	0.000294	0.001701
700	50	VarDict	-0.02473	0.030784
700	100	FreeBayes	6.59E-05	0.001007
700	100	LoFreq	-0.00042	0.001507
700	100	Mutect2	0.028357	0.091307
700	100	VarDict	-0.02184	0.029804
700	100	VarScan2	-0.0069	0.009917
700	300	Mutect2	0.105326	0.159485
700	75	FreeBayes	0.000174	0.000952
700	75	LoFreq	-0.00055	0.001577
700	75	Mutect2	-0.00919	0.069265
700	75	VarDict	-0.02179	0.028567
700	75	VarScan2	-0.0081	0.007872
1000	50	LoFreq	-0.00033	0.001058
1000	50	Mutect2	0.007706	0.06155
1000	50	VarDict	-0.02387	0.027171
1000	50	VarScan2	-0.01151	0.008783
3000	150	LoFreq	-0.00058	0.00149
3000	150	VarDict	-0.01766	0.026183
3000	150	VarScan2	-0.00465	0.008852

5000	150	LoFreq	-0.00129	0.002423
5000	150	Mutect2	0.064854	0.134471
5000	150	VarDict	-0.02505	0.030722
5000	150	VarScan2	-0.00427	0.006441
1000	75	LoFreq	-0.00039	0.00119
1000	75	Mutect2	0.008723	0.085944
1000	75	VarDict	-0.02203	0.027327
1000	75	VarScan2	-0.00803	0.007641
300	75	VarDict	-0.02164	0.026596
300	75	VarScan2	-0.00779	0.008945
5000	75	Mutect2	-0.00983	0.068195
1000	100	Mutect2	0.028322	0.098077
3000	100	FreeBayes	4.16E-05	0.000913
3000	75	LoFreq	-0.00113	0.002529
3000	75	Mutect2	0.000728	0.075262
3000	75	VarDict	-0.02467	0.031103
3000	75	VarScan2	-0.00892	0.008575
3000	150	Mutect2	0.029963	0.124928
700	300	FreeBayes	0.000218	0.00119
3000	300	VarDict	-0.0252	0.031075
3000	300	VarScan2	-0.00344	0.00933
1000	300	LoFreq	-0.00059	0.001688
5000	100	LoFreq	-0.00056	0.001319
1000	300	Mutect2	0.106307	0.152702
5000	100	Mutect2	0.006105	0.089975
1000	300	VarDict	-0.02218	0.027725
5000	100	VarDict	-0.02718	0.033707
1000	300	VarScan2	-0.00308	0.009078
5000	100	VarScan2	-0.0059	0.003611
5000	50	FreeBayes	-7.90E-05	0.001102
5000	50	LoFreq	-0.00155	0.002785
5000	50	Mutect2	0.033549	0.073311

5000	50	VarDict	-0.02952	0.031614
5000	50	VarScan2	-0.01198	0.007044
3000	300	LoFreq	-0.00069	0.001552
3000	300	Mutect2	0.103847	0.174384
3000	300	FreeBayes	-0.00029	0.003762
300	75	LoFreq	-0.0007	0.00206
3000	75	FreeBayes	9.01E-05	0.001105
5000	75	VarDict	-0.02487	0.030898
5000	75	VarScan2	-0.00797	0.006133
300	150	FreeBayes	-1.37E-05	0.000334
5000	100	FreeBayes	8.59E-05	0.001086
1000	300	FreeBayes	-1.06E-05	0.000118
300	75	FreeBayes	7.88E-05	0.000886
300	75	Mutect2	0.002589	0.064676
1000	75	FreeBayes	0.000159	0.001006
5000	300	FreeBayes	0.000145	0.00103
5000	300	LoFreq	-0.00083	0.001851
5000	300	Mutect2	0.066403	0.157371
5000	300	VarDict	-0.02602	0.030455
5000	300	VarScan2	-0.00307	0.010432
3000	50	FreeBayes	0.000102	0.001175
3000	50	VarDict	-0.02658	0.029203
3000	50	VarScan2	-0.01214	0.008314
1000	150	FreeBayes	-8.48E-05	0.000553
1000	50	FreeBayes	6.57E-06	2.40E-05
3000	50	LoFreq	-0.00088	0.002095
3000	50	Mutect2	0.009532	0.071325
1000	100	FreeBayes	0.000742	0.002482
5000	75	FreeBayes	0.000382	0.00227
5000	75	LoFreq	-0.00082	0.001689
3000	150	FreeBayes	8.73E-06	1.75E-05

5000	150	FreeBayes	-1.97E-09	2.62E-09

S7 ΔAF min max data

Caller	ΔAF min	ΔAF max
Mutect2	-0.2627472	0.8667507
Freebayes	-0.03591529	0.01597139
LoFreq	-0.01893346	0.00000051
VarDict	-0.1760923	0.01388889
VarScan2	-0.1746923	0.06010345

S8 Metrics

Regarding the equation that gives the AF deviation, $\Delta AF = AF_{Caller} - AF_{GT}$, it becomes apparent that the ideal scenario is when the AF deviation equals zero ($\Delta AF = 0$), which can be achieved when the reported AF from the callers is the same as the one of the ground truth. On the other hand, $\Delta AF > 0$ when a variant caller provides an AF that is higher compared to the real value in the ground truth. Finally, $\Delta AF < 0$ represents variants that are falsely reported with lower AF by the callers. It should be noted that this comparison can be only applied in the TP variants; in the case of the FP variants the AF of the ground truth equals zero ($AF_{GT} = 0$), while the AF of the caller is zero for FN variants ($AF_{Caller} = 0$).

To measure the callers' performance we implemented the ratio of TP against FN for each individual caller, by using the Recall (aka true positive rate and sensitivity), as shown in **Equation S8.1:**

$$Recall = \frac{TP}{TP + FN} \qquad \text{(S8.1)}$$

as well as the Precision (aka positive predictive value and specificity), which calculates the fraction of the called variants that were actually TP and is shown in **Equation S8.2**:

$$Precision = \frac{TP}{TP + FP}. \qquad \text{(S8.2)}$$

It should be noted that, within the scope of our analysis, no variants fall into the True Negative (TN) category. In our context, TN would correspond to variants absent from both the ground truth and the caller's output.

S9 Further study of ΔAF

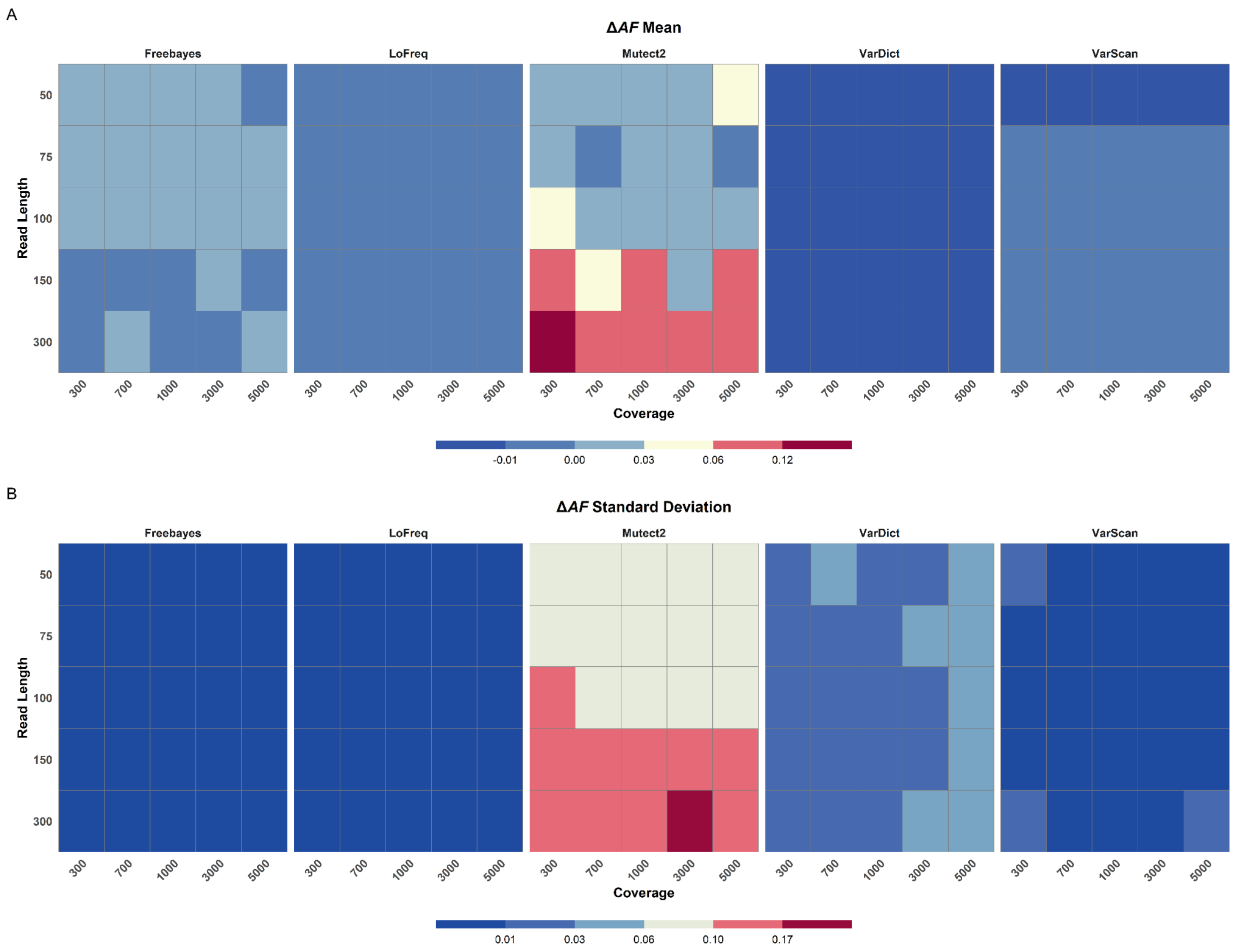

Figure S1: Heatmap showing ΔAF, as defined in Equation 3, for the SNVs True Variants analysis. Rows represent varying read lengths, and columns represent different coverage values. Colors indicate mean and standard deviation values, ranging from low (best, blue) to high (worst, red); note that the scale and range of each legend differ for better visualization. **(A)** Mean deviation values are shown, and **(B)** standard deviation values are depicted.

We evaluated how accurately the callers quantified each variant in the SNVs True Variant analysis, focusing specifically on the precision of their reported AF. To this end, we defined a new metric, the AF deviation (ΔAF), which quantifies the difference between the reported AF and the ground truth. As shown in **Figure S1A**, LoFreq and VarDict consistently demonstrated the most stable and accurate performance, with a near-zero mean error across all combinations of read length and coverage. The uniform dark blue color of their respective heatmaps indicates a high degree of accuracy and robustness to changes in sequencing parameters. In stark contrast, Mutect2 showed the greatest deviation in its mean AF estimation, showing a significant positive bias (overestimation) at longer read lengths, particularly for lower coverage values. It is also worth noting that Mutect2 had the highest ΔAF dispersion, by reaching both the highest values and the lowest values (0.87 and -0.26, respectively). On the contrary, LoFreq exhibited the best performance with its highest value being 5.1×10^{-7} and the lower -0.019, as shown in **Section S7** of the Supplementary Material. FreeBayes and VarScan2 performed generally

well, but with slight biases (FreeBayes for shortest reads and VarScan2 for longer reads), suggesting less consistent accuracy compared to VarDict and LoFreq.

The ΔAF standard deviation is depicted in **Figure S1B**. FreeBayes, LoFreq and VarScan2 showed consistently low standard deviations across the entire feature space. Conversely, Mutect2 displayed the highest standard deviation, particularly for higher read lengths and coverages. VarDict also showed some variation, but its standard deviation remained lower than that of Mutect2 across all conditions.

S10 Base quality exploration

To evaluate the effect of sequencing quality variability on variant detection, an additional set of datasets was generated by modifying the base-quality distributions of the synthetic reads to better resemble realistic sequencing conditions. The same benchmarking workflow was subsequently applied and the resulting variant classifications were compared against those obtained from the original datasets.

The introduction of realistic base-quality variability produced distinct caller-specific effects. While FreeBayes and LoFreq showed reduced sensitivity and Mutect2 remained largely stable, VarDict and VarScan exhibited increased sensitivity, with VarDict demonstrating the largest overall change. In all cases, false positive counts were affected less substantially than true positive and false negative classifications. The results are shown in **Figure S2**.

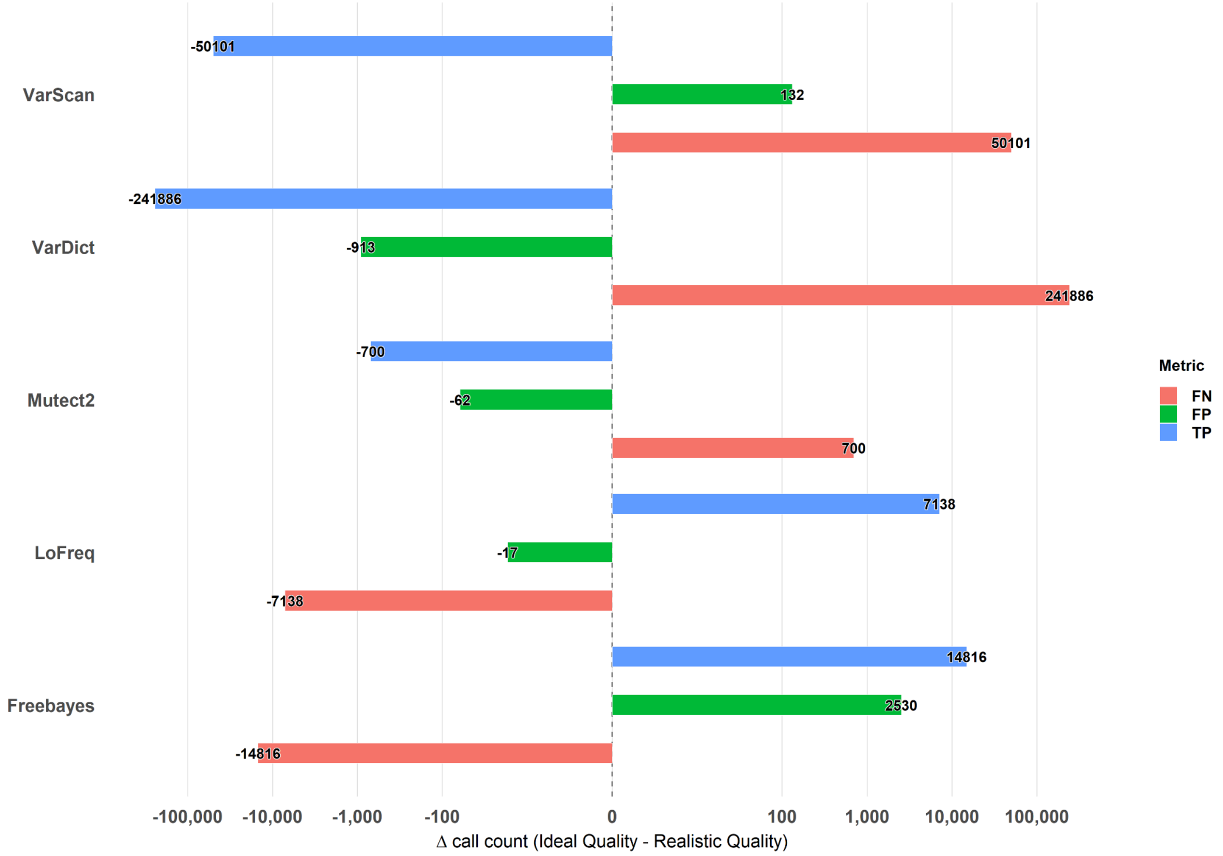

Figure S2: Effect of Realistic Sequencing Quality Profiles on Variant Calling Outcomes. Change in variant classification counts following the introduction of realistic base-quality profiles. Bars represent the difference in call counts between the ideal-quality and realistic-quality datasets (Δ=Ideal Quality−Realistic Quality). Positive values indicate fewer calls in the realistic-quality datasets, whereas negative values indicate more calls in the realistic-quality datasets. The x-axis is displayed on a pseudo-logarithmic scale to facilitate visualization of both small and large changes. TP: true positives (blue bars); FP: false positives (green bars); FN: false negatives (red bars).

S11 GIAB Benchmark Variant Recovery Analysis

To further explore the relationship between the synthetic benchmarking framework and experimentally derived biological variants, an additional analysis was performed using two independent GIAB benchmark samples: HG001 (NA12878) and HG002 (NA24385). High-confidence variants located within the TP53 genomic locus were extracted from the corresponding GIAB truth sets and compared against the variants represented in the Synth4Bench datasets.

A total of 10 exact variant matches were identified for HG001 and 8 exact variant matches were identified for HG002. These variants were subsequently tracked across the synthetic dilution framework and evaluated according to whether they were successfully recovered by each variant caller.

Recovery rates were calculated as the proportion of GIAB-matched variants successfully detected across all Synth4Bench datasets. Results are presented in the following table. Consistent recovery trends were observed between the two GIAB samples, with LoFreq and VarScan showing the highest recovery rates (54.0–56.4%), followed by VarDict (46.0–50.6%), while Mutect2 and FreeBayes exhibited lower recovery rates (23.8–29.5%).

Recovery rates of GIAB benchmark TP53 variants across variant callers.

**Callers**	**HG001 Recovery (%)**	**HG002 Recovery (%)**
Freebayes	23.8%	25.0%
Mutect2	25.7%	29.5%
LoFreq	54.0%	56.4%
VarDict	46.0%	50.6%
VarScan	55.9%	56.4%

GIAB HG001 (NA12878) TP53 variant information.

**Genomic Position**	**REF**	**ALT**
chr17:7679909	C	T
chr17:7676483	C	G
chr17:7676154	C	G
chr17:7675519	T	C
chr17:7675327	G	A
chr17:7674797	A	G
chr17:7672246	A	G

chr17:7671618	G	A
chr17:7671461	G	A
chr17:7671457	G	A

GIAB HG002 (NA24385) TP53 variant information.

Genomic Position	REF	ALT
chr17:7678265	C	T
chr17:7676483	C	G
chr17:7676154	C	G
chr17:7675519	T	C
chr17:7675327	G	A
chr17:7674797	A	G
chr17:7672246	A	G
chr17:7671626	C	T

S12 Modeling low-frequency variants

In an individual BAM file of $coverage_i$ and allele frequency f_i let us consider that a variant is present in a number of reads, denoted $reads_i$. This number of reads is fixed throughout the process of merging (those present in the individual file remain the same in the final merged file) and thus we treat it as constant, i.e. $reads_i = const$. After the merging process, we end up with a merged BAM file of $coverage_m$ and a new allele frequency F for the same variant. These two allele frequencies are given by equations:

$$\begin{aligned} f_i &= \frac{reads_i}{coverage_i} \\ F_m &= \frac{reads_i}{coverage_m} \end{aligned} \tag{S12.1}$$

which if we solve for $reads_i$ and take into account that $coverage_m = 10 \cdot coverage_i$ (because 10 individual BAM files are combined to give the final merged BAM file), become:

$$\begin{aligned} reads_i &= f_i \cdot coverage_i \\ reads_i &= F_m \cdot 10 \cdot coverage_i \end{aligned} \tag{S12.2}$$

after a few simple calculations these eventually give the AF of the variant in the final merged BAM file:

$$F_m = \frac{f_i}{10}. \tag{S12.3}$$

So far we have treated the merging process from an ideal lens, without taking into account any other reads that might be present in some other individual BAM files supporting the same variant in the same genomic position. In reality, there is usually an extra fixed number of reads denoted as $reads_{overlap}$, from overlapping reads, that add up in the final merged BAM file. Given this Eq. S12.2 now becomes:

$$\begin{aligned} reads_i &= f_i \cdot coverage_i \\ reads_i + reads_{overlap} &= F_m \cdot 10 \cdot coverage_i \end{aligned} \tag{S12.4}$$

and given that $coverage_m = 10 \cdot coverage_i$, it eventually becomes:

$$F = \frac{f_i}{10} + \frac{reads_{overlap}}{coverage_m} \tag{S12.5}$$

the first allele frequency component is the same as in the ideal scenario shown in Eq. S12.3 and the second component is from the rest overlapping reads, i.e. the allele frequency from overlap $F_{overlap} = \frac{reads_{overlap}}{coverage_m}$. So the final allele frequency for that variant, taking into account overlapping reads from other sources, becomes:

$$F = F_m + F_{overlap}. \tag{S12.6}$$
